# Entangled ZnO on Ultrathin Hollow Fibers for UV-Aided Pollutant Decomposition

**DOI:** 10.1021/acsami.1c21554

**Published:** 2022-02-21

**Authors:** Xi Wang, Shaojun Xu, Evelyn Chalmers, Xiaogang Chen, Yong Liu, Xuqing Liu

**Affiliations:** † Department of Materials, School of Natural Sciences, The University of Manchester, Oxford Road, Manchester M13 9PL, U.K.; ‡ UK Catalysis Hub, Research Complex at Harwell, Didcot OX11 0FA, U.K.; § Cardiff Catalysis Institute, School of Chemistry, Cardiff University, Cardiff CF10 3AT, U.K.; ∥ School of Textile, Tiangong University, No. 399 Bin Shui Xi Road, Xi Qing District, Tianjin 300387, P. R. China

**Keywords:** curcumin interfacial functionalization, electroless deposition, zinc oxide, natural hollow fiber substrate, wastewater

## Abstract

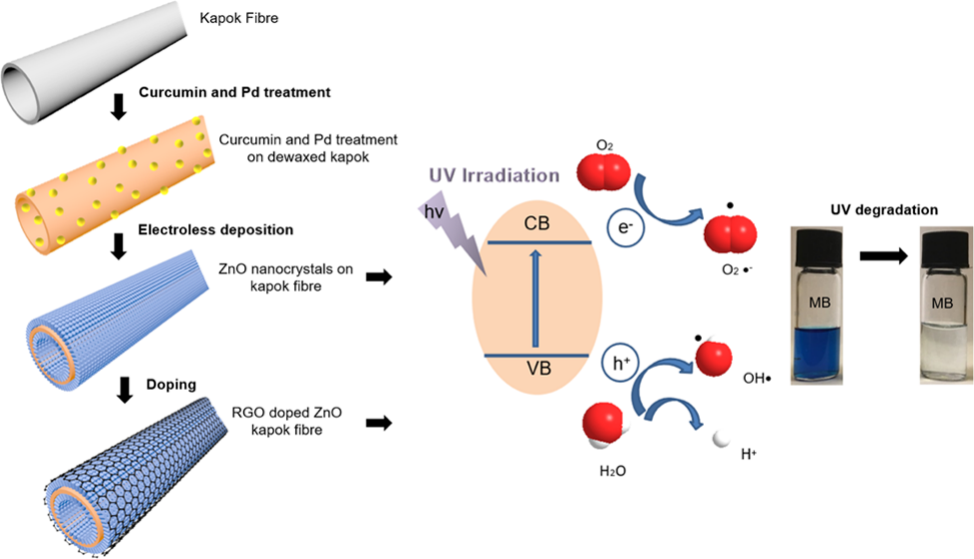

Zinc oxide (ZnO), a widely used ultraviolet (UV) degrading substance, offers high selectivity for wastewater treatment, but the leaching of ZnO into water could cause secondary contamination. Using porous substrates to fix and load ZnO is a promising technical method to improve the water purification efficiency and recycling durability of ZnO. However, limited by the slow kinetics and shielding effects, it is challenging to use traditional techniques to introduce ZnO into the interior of a hollow structure. Here, inspired by an ancient dyeing procedure, we formed a unique single-molecule bio-interfacial entanglement as an absorption layer to capture the catalyst for ZnO electroless deposition (ELD) on the surface of natural ultrathin hollow-structured Kapok fibers. With curcumin serving as a linking bridge, ELD allowed the spontaneous formation of intensive ZnO nanocrystals on both the outer and inner walls. ZnO-kapok as the catalyst for ultraviolet photodecomposition of organic pollutants (methylene blue (MB) and phenol as model pollutants) delivered a decomposition efficiency of 80% and outstanding durability. Further modification of the ZnO-kapok catalyst by doping with reduced graphene oxide (rGO) showed an improvement in photodegradation performance of 90% degradation under 2-h irradiation with 21.85 W/dm^2^ light power. Moreover, to the best of our knowledge, this is the first report featuring ZnO loading on both the outer and inner walls of a fiber-structured hollow kapok material, which provides inspiration for immobilization of metallic oxides on hollow-structured materials for further applications in renewable catalysis, chemical engineering, and energy storage fields.

## 1 Introduction

In recent years, increasing water-soluble organic pollutants have seriously influenced the environment, due to the pollutants from textile, paper, or other industrial manufacturing. The release of polluted water into the ecosystem would be a dramatic disaster for aquatic life. Hence, the pollution removal system in aqueous solutions requires emerging development. There have been a growing number of efforts focusing on water pollution control by reducing contamination through both the adsorption process and the chemical coagulation technique.^1−4^ However, traditional adsorption techniques can only transfer organic pollutants from water to solid substrates, which will introduce secondary pollution and require further treatment.^5^ Advanced trends in the field of environmental pollution control have led to growing interest in photocatalysis. In addition to this, using absorbents to collect pollutants is considered to be the most effective and economical strategy. Establishing a durable structure can be essential to create an eco-friendly water purifier as an effective solution for reducing aqueous effluents, especially those from textile industries.

Photocatalysis is an eco-friendly approach for pollutant degradation by the application of solar energy to activate the catalytic process under mild reaction conditions.^6−9^ Various photocatalysts, especially semiconductors, including zinc oxide (ZnO),^10−19^ titanium oxide (TiO_2_),^20,21^ tin oxide (SnO_2_),^22^ zirconia (ZrO_2_),^23^ and copper oxide (CuO)^24^ have been broadly investigated for their role as sensitizers for the light-induced redox process. Among these metal oxides, ZnO is a nontoxic and relatively cost-effective n-type semiconductor with a wide band gap of 3.37 eV and high binding energy of up to 60 MeV.^25^ More importantly, it is a tunable material and sensitive to UV light, whose photocatalytic performance can be increased by doping with metals or other materials.^26−31^ In previous research, zinc oxide nanocrystals have been widely used in the area of water pollutant disposal, used as UV-blocking materials, piezoelectric materials, etc.^15−17,32,33^ However, the recyclability of ZnO nanocrystals has not achieved the expectation and the nano-powder catalyst will also introduce secondary pollution. In addition, nanocrystals dispersed in water can lead to conglobation easily and reduce the effective surface area, which can decrease the reaction efficiency of photocatalytic degradation.

The high-surface-area natural hollow fibers with effectively designed and controlled morphology and functional properties are emerging as alternatives to the conventional absorbent and catalyst substrates in sustainable chemistry. Compared to the conventional substrates (such as polyimide membranes,^34−36^ silicon wafers,^37^ glass,^38^ or ceramics^39^), natural fibers can significantly reduce the secondary contamination from used heterogeneous catalysts, especially, in the liquid-phase processes. For example, during water pollution removal by reducing the contamination, the metal oxide (like ZnO) based heterogeneous catalysts used for the photodecomposition technique could lead to nanocrystal exfoliation in water causing heavy metal/solid pollution.^40−47^

The natural fibers, kapok (*Ceiba pentandra*) fibers, are one of the most sustainable fibers in the market today leaving no human footprint behind, which are common seed fibers in southern Asia and the East Indies with more than 86% of hollowness.^48,49^ Also, the kapok fiber is very cheap due to its short fiber length without spinning value, but its high porosity with a high specific area provides great potential as the absorbent and catalyst substrate.^50−54^ For example, making full use of natural kapok fibers as substrates is an ideal approach for fabricating supported UV-degradation catalysts. Particularly, the ultrathin wall of the kapok fiber substrate allows ultraviolet (UV) light to pass through smoothly, which will further enhance the efficiency of UV degradation.^55−58^

Despite the great potential of high-surface-area natural hollow fibers, the organic nature of the fibers with waxing/hydrophobic surfaces makes it very challenging to act as absorbents in the liquid phase or substrate to establish metal–support interactions as that in conventional inorganic heterogeneous catalysts. Moreover, the stability of the loaded nanoparticles over the fibers will be reduced during the reaction.^59^ The method of grafting polymer as an adhesion layer was studied for deposition of metal or metal oxides over the fibers to improve the selectivity and uptake efficiency of the metal or metal oxides over the catalyst.^32^ However, the preparation method of polymer brushes is complicated and requires N_2_ protection. Therefore, a simple method for preparing a biodegradable interfacial layer with high activity is critically required, but so far there are comparatively few studies on the surface functionalization and modification of the natural hollow fibers.

In this study, inspired by the dyeing process, a simple fabric impregnation method was used to functionalize ultrathin, hollow kapok fibers with curcumin, a plant polyphenol used for dyeing and decorating, providing a uniform and dense coating on the bio-substrate with strong adhesion.^60^ Facilitated by a simple electroless deposition (ELD) technique, the nanoparticles can be controlled during in situ growth over the inner and outer walls of ultrathin, hollow kapok fibers. Here, typical ZnO as active sites for UV-degradation catalysts was used as model nanoparticles. Then, the synthesized ZnO-loaded ultrathin, hollow kapok fibers as hybrid UV-degradation catalysts were evaluated as a proof-of-concept for ultraviolet photodecomposition of organic pollutants with methylene blue (MB) and phenol as model pollutants. The effect of morphologies of ZnO nanocrystals on the photodegradation performances was studied. To reveal the durability, 5 recycle photodegradation tests toward the typical dye pollutant were conducted. Furthermore, the effect of rGO doping deposition on the efficiency of hybrid UV-degradation catalysts was also investigated.

## 2 Experimental Section

### 2.1 Materials

Lipase and zinc nitrate hexahydrate were purchased from Thermo Fisher Scientific (U.K.). Disodium monohydrogen phosphate, sodium dihydrogen phosphate, ethanol, curcumin, ammonium tetrachloropalladate, dimethylamine borane (DMAB), methylene blue (MB), and phenol were purchased from Sigma-Aldrich (U.K.). Graphene oxide (GO) was synthesized in our lab (shown in the Supporting Information). All chemicals were used as received. Kapok fibers and cotton fibers were picked in Nanning, China.

### 2.2 Fabrication of ZnO-Loaded Kapok Fibers

**Figure 1:**
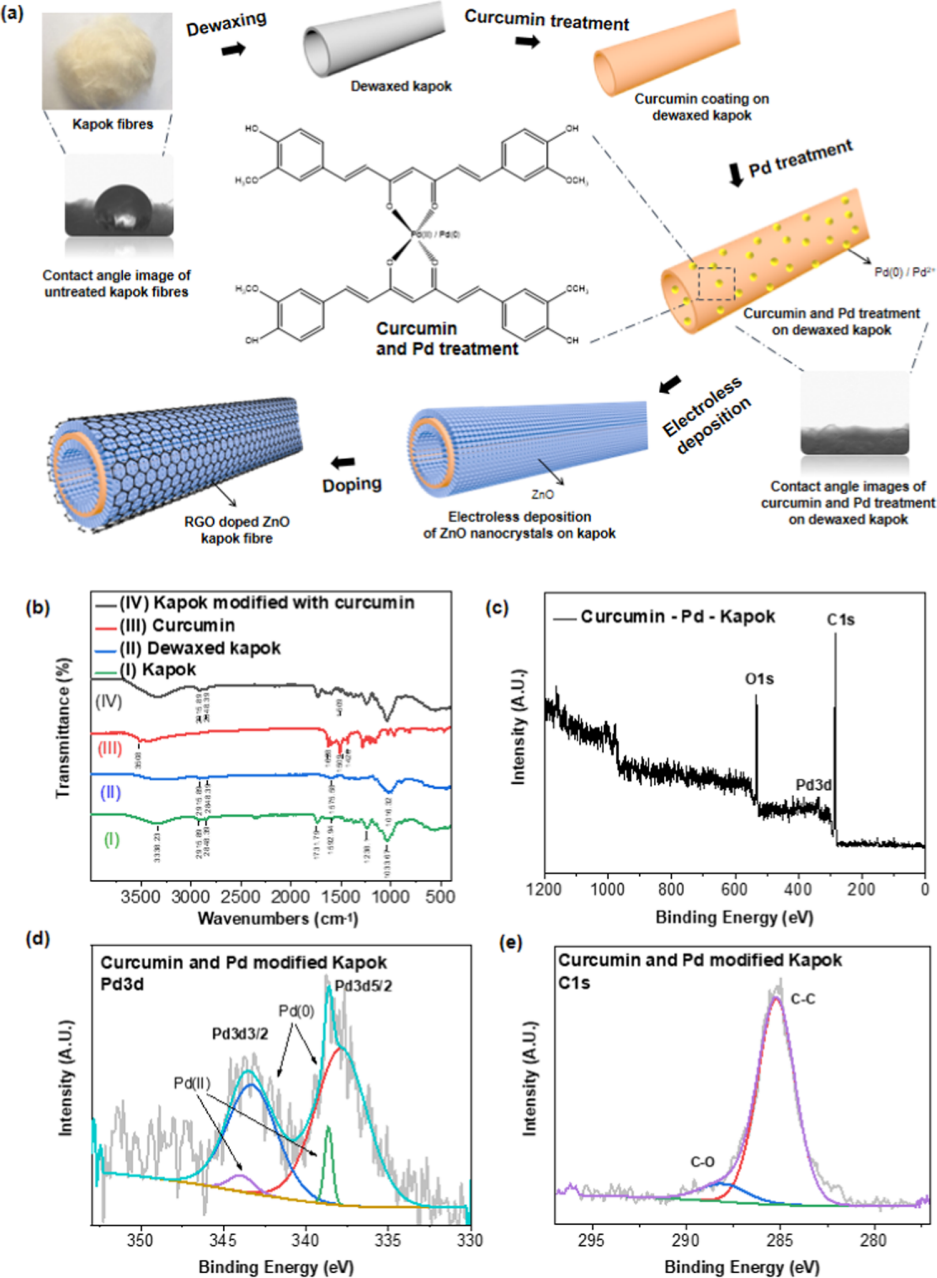
Preparation and characterization of the ZnO nanocrystals loaded on natural hollow kapok fibers. (a) Process of obtaining a ZnO nanocrystal-loaded hollow kapok fiber by anchoring Pd catalysts by curcumin bio-interfacial layer. (b) FTIR spectra of dewaxing and interfacial curcumin pre-treatment of the fiber. (c) XPS spectra of curcumin and Pd-modified kapok fibers, and (d) Pd 3d and (e) C 1s of the sample.

As shown in Figure 1a, nanostructured zinc oxide was grown on curcumin-pretreated kapok fibers by the ELD process. First, the dewaxed fiber specimens (the dewaxing process shown in the Supporting Information) were immersed in 0.5% curcumin ethanol solution for 12 h denoted as curcumin-pretreated kapok (CK). Then the samples were rinsed with DI water and dried at 40 °C. Subsequently, by immersing the specimens in 0.005 M ammonium tetrachloropalladate solution for 30 min, Pd^2+^ was deposited on the fibers by chelation as a layer for the following ELD process. The fibers were rinsed in ethanol solution to remove organic impurities and then in DI water. Then, 50 mM zinc nitrate hexahydrate and 50 mM DMAB solution were mixed as an electroless plating bath. The Pd^2+^-loaded fibers were placed in the plating solution at 90 °C for 5, 10, 30, 60, 90, and 120 min. Then, the samples were rinsed with DI water three times to remove physically absorbed ZnO. In the end, the specimens were dried at 40 °C for 12 h, which were denoted by curcumin-pretreated kapok fibers loaded with ZnO (CKZ-*X*, *X* represent the prolonged ELD time).

### 2.3 Doping of Reduced Graphene Oxide on ZnO-Loaded Fibers

To dope reduced graphene oxide, 10 g of CKZ samples with different prolonged times were dipped into 20 mL of 8 mg/mL graphene oxide aqueous solution for 2 h while stirring. Later, the specimens were transferred into an oven heated at 400 °C for 1 h protected by argon gas for reduction of GO.

### 2.4 Characterization of ZnO Composite UV Degradation Catalysts

Morphology and crystalline structure of supported UV-degradation catalysts were determined by scanning electron microscopy (SEM) using a Zeiss Ultra 55 microscope (accelerating voltage 3 kV), by transmission electron microscopy (TEM) using an FEI Tecnai G2 20 (LaB6) microscope, and by X-ray diffraction (XRD) using a PANalytical (Philips) X’Pert Pro X-ray diffractometer. XRD patterns were recorded at a scanning rate of 0.033° step at 530 s/step, accelerating voltage of 40 kV, and anode current 40 mA in the 2θ range from 4 to 80° with a copper tube X-ray source (Cu Kα, λ = 0.154 nm). Each sample was coated with a layer of Pt prior to observation by SEM and energy-dispersive X-ray detector (EDX). The hydrophilicity of the enzyme and curcumin-pretreated fibers was demonstrated on a DSA100 contact angle goniometer and the organic functional group changes were observed using a Bruker Vertex 80 Fourier transform infrared spectroscopy (FTIR). The immobilization of Pd on the surface of fiber substrates was determined using an X-ray photoelectron spectrometer (XPS) with 15 kV anode voltage, 80 eV pass energy, 10 mA current, 1486.7 eV photons, monochromated AI Kα X-ray source, and 500 ms constant dwell time under ultrahigh vacuum conditions. The loading of ZnO was measured by the Thermogravimetric analysis (TGA) on a Perkin-Elmer DSC7 system under N_2_ conditions. To account for rGO doping, the Raman spectra of the samples were measured with a Renishaw RM1000– 514 nm Raman system with a 1 μm laser spot size and below 10 mW power. Electron paramagnetic resonance (EPR) measurements were carried out on a Bruker EMXmicro EPR spectrometer equipped with a Bruker ER4122-SHQ resonator. Spectrometer settings were microwave power of 23 dB (1.1 mW), modulation amplitude of 0.5 G, sweep time of 60 s, and a receiver gain of 30 dB with an average microwave frequency of 9.86 GHz.

### 2.5 Investigation of Photodegradation Performance and Durability

In the UV irradiation experiments, a 230 V, 50 Hz, and 0.95 A high-intensity ultraviolet lamp (λ = 365 nm, SPECTROLINE Modle SB-100P/FB) was used as the UV irradiation source; the distance between the light source and the specimens was ∼20 cm. The light power was 21.85 W/dm^2^. To test the photodegradation performance, beakers were used as the container for pollutant solution. As the beakers do not have lids, it will not interrupt the absorption of UV light by the samples. For each glass bottle, 10 mL of 0.1 mM methylene blue aqueous solution and 0.05 g of dried ZnO-loaded kapok fibers were added. The solution was not agitated. Due to the hollowness of the fibers, the samples were floating on the solution in the container. Meanwhile, 3 contrast groups were prepared: one beaker with pure MB solution, one beaker with MB solution with raw kapok, and another beaker with MB solution with ZnO powder. Then all containers were kept in a dark environment for 30 min to reach a stable equilibrium. Subsequently, the mixtures were under UV irradiation for 0.5, 1, 1.5, 2, 4, and 5 h, respectively. The rGO-doped samples were treated by the same procedures to investigate the decomposition performance after reaching the balance in the dark environment. UV–vis measurements were performed to measure the degradation process of methylene blue in the solutions using an ultraviolet–visible spectrometer. Afterward, the samples were washed for up to 5 cycles using a phosphor-free laundry detergent without enzyme (ECE Formulation Standard detergent) and their photodegradation performance toward MB was modified after each washing cycle for the reusability test. The morphology and photodegradation properties of ZnO kapok fibers after washing cycles were investigated to reveal their stability and durability.

## 3 Results and Discussion

### 3.1 Interfacial Modification ZnO-Loaded Fiber-Based UV Degradation Catalysts

Figure 1a presents a study concept of the fabrication process. The kapok fiber, with a natural hollow structure that can offer a high specific area (shown in Figure S1a), is considered an ideal bio-substrate for loading the UV-degradation catalyst to fabricate an efficient supported UV-degradation catalyst. Also, the unique structure of kapok fibers can assist in pollutant collection from the aquatic environment to improve the entire water purification process due to its absorption ability. In Figure 1a, the fabrication started with an eco-friendly enzyme dewaxing process of natural kapok fibers, as the fibers present hydrophobic properties due to a layer of wax covered on the surface to prevent moisture and mildew. The contact angle of raw kapok fibers was around 151°, as shown in Figure 1a. Then, the dewaxing process was used to prepare the flexible fiber substrate with a hydrophilic surface for later modifications. Moreover, inspired by the dyeing process, curcumin was coated on the dewaxed fibers stubbornly via hydrogen bonds to endow the fiber with many more coordination sites. After the dewaxing and curcumin treatment process, the hydrophilic surface of fibers was ready for later catalyst loading and its contact angle, shown in Figure 1a and Video S1, had decreased to 0°.

The evolution of the FTIR spectra of raw kapok fibers, dewaxed kapok fibers, curcumin, and curcumin-pretreated kapok fibers is shown in Figure 1b to confirm the dewaxing process and the coating of curcumin. After the dewaxing process, the position of characteristic peaks of cellulose fiber did not change obviously, which suggests that the dewaxing process has no significant effect on the chemical composition of cellulose. The absorption band at 3338.23 cm^–1^, associated with the stretching vibration of −OH in cellulose of kapok fibers, was observed to be reduced in Figure 1b(II). It indicates the breaking of hydrogen bonds transferred into free cellulose hydroxyl groups.^61,62^ The characteristic peaks of acetyl (CH_3_–C(O)−) in lignin of kapok fibers were observed at 1731.79 and 1238.1 cm^–1^, which had a significant decrease after the dewaxing process. This is attributed to the desertification of lignin in kapok fibers.^62^ The bands at 1592.94 cm^–1^, assigned to the stretching vibration of C–C in various substituted aromatic rings in lignin of kapok fibers, decreased due to the cleavage of the aromatic ring in lignin. These results have suggested effective removal of wax from kapok fibers. After immersion in curcumin solution, the fiber specimen combined the characteristic peaks of curcumin at 1428 cm^–1^ attributed to C=C aromatic stretching and those at 1509 cm^–1^ due to the mixed vibrations (carbonyl bond stretching vibrations ν(C=O), in-plane bending vibrations of aromatic δ CC–H of keto and enol configurations, and in-plane bending vibrations of aliphatic δ CC–C, δ CC=O, and stretching vibrations of aromatic ν CC bonds of keto and enolic form of curcumin) with cellulose characteristic peaks.^63^

Afterward, as the Pd ions can be immobilized on the fiber substrate by the cation−π interaction with benzene groups in curcumin, the curcumin-modified samples were immersed in the ammonium tetrachloropalladate solution to anchor Pd as a catalyst and as growth sites for the subsequent ELD process to be triggered on the polyphenol interfacial layer.^64^ In Figure 1d, the Pd 3d XPS spectrum shows two spin–orbit doublets: one is for Pd(0) at binding energies of 337.9 and 343.3 eV and the other is for Pd(II) at binding energies of 338.6 and 343.9 eV. The C 1s XPS spectrum in Figure 1e indicates the existence of C–O and C–C compounds. These results suggested the reduction of part of palladium during the chelation of Pd ions. In addition, the EDX images (in Figure S1b–f) have revealed the surface chemical composition, which showed an even spreading of Pd. Thus, the pretreatment process would provide a uniform linkage layer between the fiber substrate and functional material. Additionally, to further illustrate the influence of the modified curcumin layer in the growth of ZnO on fibers, we prepared some control samples without curcumin pretreatment. As shown in Figure S2, the ZnO nanostructures were distributed unevenly throughout the kapok fibers without curcumin modification and showed random morphologies, which can be due to the complexity of natural materials and inadequate growth sites. The control groups have suggested that the modification with curcumin can provide a platform with multiple growth sites for uniform ZnO nanocrystal deposition.

### 3.2 Characterization of ZnO-Loaded Fiber-Based UV Degradation Catalysts

**Figure 2:**
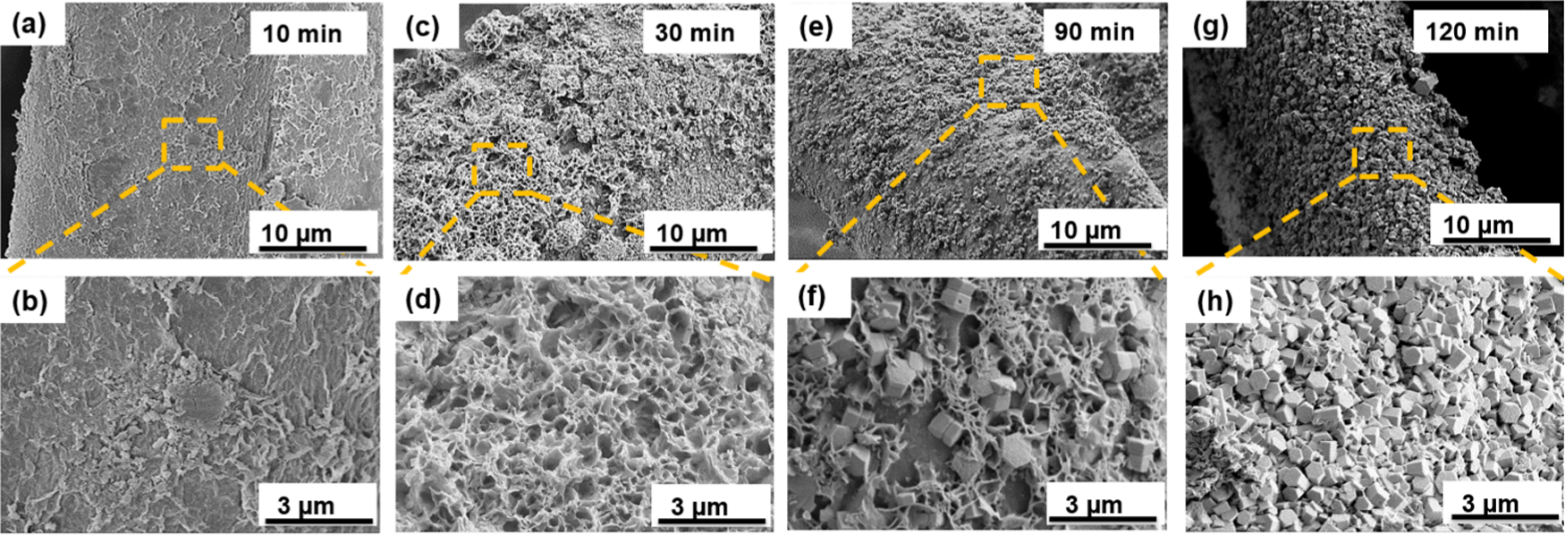
Effect of growth time on the morphology of ZnO nanoparticles deposited on the kapok fiber substrate, which is pretreated with curcumin and ammonium tetrachloropalladate by an electroless deposition process. Morphology analysis of ZnO nanocrystals obtained after (a, b) 10 min, (c, d) 30 min, (e, f) 90 min, and (g, h) 120 min prolonged times.

ZnO nanocrystals can be synthesized on Pd catalytic sites via ELD. In this study, the ELD time would be optimized from 0 to 120 min at 90°C. The SEM images (shown in Figures 2a–h and S3) reveal the generation of ZnO on the surface of fibers and illustrate the morphology changes during the growth process. Upon immersing the modified fibers in zinc nitrate (Zn(NO_3_)_2_) and dimethylamine borane (DMAB) solution, the fiber substrate surface was immediately covered with a densely distributed granular zinc seed layer, as shown in Figure 2a,b, which would assist zinc oxide nanocrystals to grow on the fibers with a compact and uniform crystal structure. The dimethylamine borane (DMAB) solution was used as a reductant in this electroless deposition, which can improve the reduction of NO_3_^–^ ions to raise the pH. The local pH increase by the reduction reaction is a key factor that can lead to the precipitation of Zn ions in this electroless deposition process. Then, granular ZnO gradually formed aggregates, some of which became adhesion flakes after a 30-min treatment. The flakes started stacking and the structure of ZnO exhibited a hexagonal close-packed (h.c.p.) phase with a hexagonal wurtzite morphology, which would be further discussed in the XRD section. Most crystals of CKZ-120 min were in the shape of hexagonal wurtzite on the outer wall of fibers with a diameter of around 200 nm. According to the classical nucleation theory and dynamics mechanism of crystal nucleation and growth, small ZnO crystal nuclei were formed spontaneously in the supersaturated reaction solution and when the crystal size reached over the critical nucleus, it would form a stable bulk phase.^65^ Therefore, the amount of the ZnO nanocrystals on fibers was increasing with the time of the ELD process expanded from 5 to 120 min and reaching a balance at around 120 min. According to the N_2_ adsorption–desorption isotherms shown in Figure S4, the deposition of ZnO nanocrystals throughout the hollow fibers can prevent general agglomeration and provide a higher specific surface area with activation sites, which can lead to a distinctive improvement of UV degrading ability after the ELD process.

**Figure 3:**
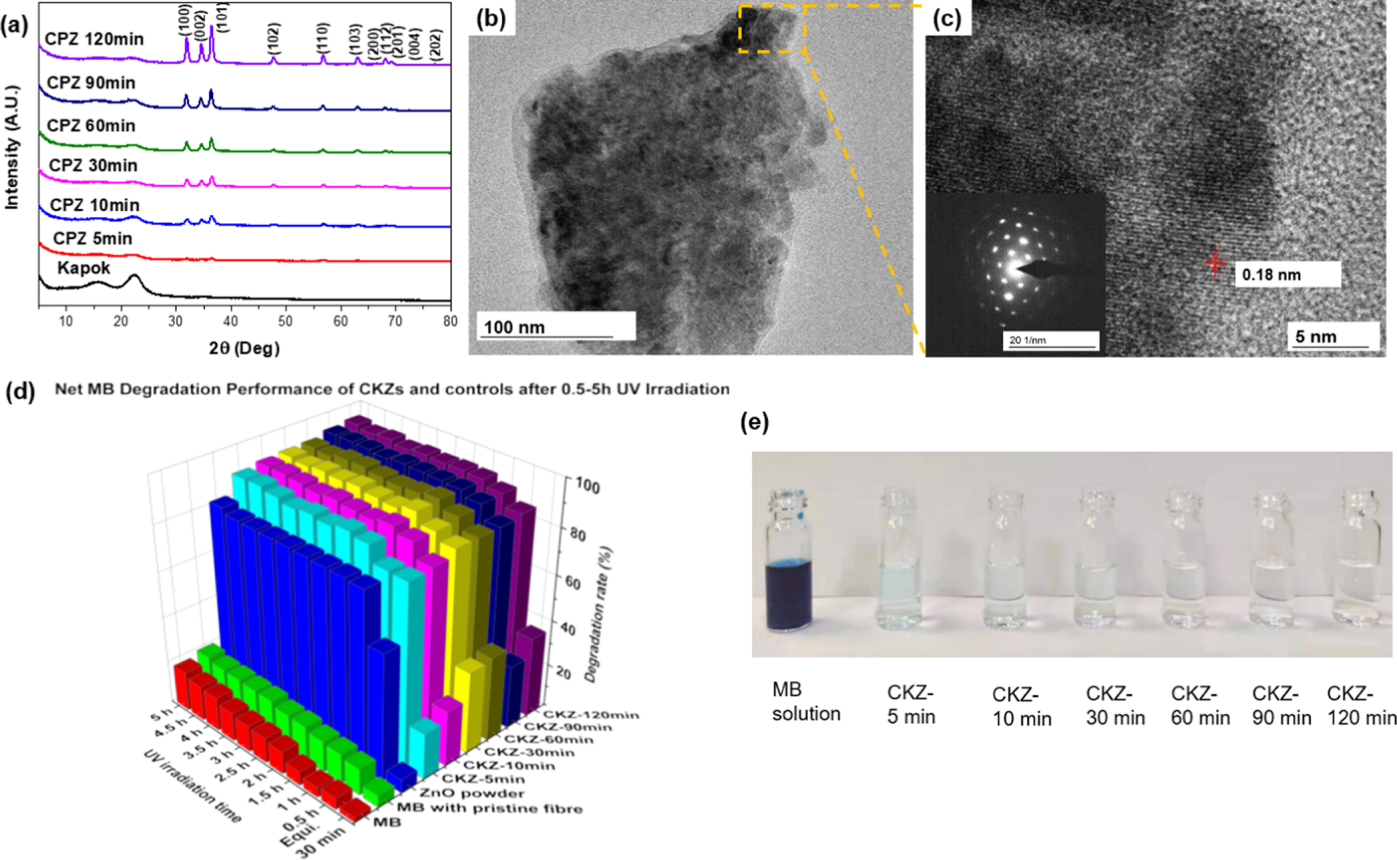
Characterization and comprehensive analysis of MB degradation efficiency. (a) XRD spectra of all ZnO-loaded kapok CKZ-5 min, CKZ-10 min, CKZ-30 min, CKZ-60 min, CKZ-90 min, CKZ-120 min, and untreated kapok fiber samples. (b) TEM image of the crystal structure of CKZ-120 min. (c) HRTEM image of the crystal structure of CKZ-120 min (inset: SAED image). (d) MB absorption rates of CKZs and control groups after 30 min of the dark absorption-equilibration experiment and net MB degradation rate of CKZ-5 min, CKZ-10 min, CKZ-30 min, CKZ-60 min, CKZ-90 min, and CKZ-120 min, and control groups, respectively, after 0.5, 1, 1.5, 2, 4, and 5 h of the UV irradiation process. (e) Digital images of pure MB solution and MB treated by CKZs after a 5-h UV irradiation process.

The crystalline structure and size of zinc oxide nanomaterials were monitored by X-ray diffraction (XRD). In Figure 3a, the XRD patterns of ZnO-loaded kapok fibers can be indexed to ZnO with the hexagonal crystal system (JCPDS no. PDF75-0576).^66^ The diffraction peaks at 2θ values of 31.9, 34.3, 36.2, 47.6, 56.6, 62.8, 67.8, and 69.2° correspond to the (100), (002), (101), (102), (110), (103), (112), and (201) planes of ZnO, while the diffraction peak at 22.3° corresponds to the typical cellulose (200) planes from kapok fibers.^67,68^ Due to the Gibbs free energy minimization principle during the crystal growth process, the anisotropy of ZnO can lead to a minimization of the internal energy of the system to contribute to crystals in preferred orientation growth. The results can also be influenced by the crystal type of the substrate to a certain extent since crystal orientation is affected by interface energy, grain priority growth direction, and interface strain energy.^69^ In general, the products show the wurtzite structure of ZnO (hcp). As the XRD peaks of zinc oxide nanocrystals became sharper along with increasing reaction time, the unit cells became more perfect crystals. The average ZnO crystallite sizes for CKZ-5 min to CKZ-120 min samples were calculated by the Scherrer equation, and were 5.56, 7.36, 11.15, 12.61, 13.15, and 15.22 nm, respectively. With increased synthesis time, the average crystallite size and final crystal size became larger during the electroless deposition period, which proves that the growth of ZnO nanocrystals with different sizes can be controlled by prolonging the time. Additionally, the ZnO remained nanoscaled crystals and abundant oxygen holes existed on the surface, which can significantly increase the reaction points. Also, the electron–hole recombination probability can be decreased due to the nano-sized crystals, which will improve the separation effect to achieve a higher photodegradation activity. The TEM and high-resolution TEM (HRTEM) images of CKZ-120 min with structural flaws are shown in Figure S5. The crystallinity of ZnO can be reduced as a result of oxygen vacancies reducing anion–cation coordination. To further demonstrate the presence of oxygen holes on the surface of nanoscaled ZnO, photoluminescence (PL) spectroscopy was utilized to analyze the oxygen vacancy in the wavelength range of 300–800 nm using a 310 nm excitation wavelength. Figure S6 illustrates the typical strong band at 390 nm and the broad absorption band at 550 nm, as well as the presence of a shoulder (470 nm), which is induced by oxygen vacancies and other interstitial defects in the ZnO nanocrystals.^70^ The oxygen vacancies would be critical in reducing the photogenerated electron and hole pair recombinations during the photocatalytic degradation reaction, consequently improving degradation performance.

Figure 3b,c shows transmission electron microscopy (TEM) images and high-resolution (HRTEM) image of the CKZ-120 min ZnO crystal. The TEM sample was prepared by immersing CKZ-120 min ZnO kapok fibers into ethanol solution under ultrasonication for 6 h, in which a small amount of ZnO crystals fell out of fibers. The TEM images of a ZnO nanocrystal further confirm that ZnO has a crystal structure composed of grains, whose diameter was around 200 nm and the result is consistent with the above SEM data. The interplanar distance that corresponds to the (101) plane of ZnO was around 0.18 nm. The inset image in Figure 3c is the selected area electron diffraction (SAED) pattern of the CKZ-120 min, which indicates that this sample was crystalline with a wurtzite structure (hcp), which agrees with the above XRD results. The shape and dimensions of the nanostructured ZnO exhibited a significant effect on the photodegradation performance, from both the specific surface area and the light-trapping ability aspects, including the ability to interact with target pollutants. Complete hexagonal wurtzite ZnO nanocrystals have a large specific surface area, particularly when grown on a substrate containing both inner and outer surface walls. As a result, these ZnO nanocrystals can not only enhance light-trapping ability but also increase the accessibility of pollutants to the ZnO surface, hence increasing light absorption independent of incidence angle, which is advantageous for sunlight-driven applications. After a 120-min growth process, the nanocrystals can serve as building blocks for whole effective architecture of the water purification system.

### 3.3 Photodegradation Activity

The ZnO-loaded fiber composites, with large crystal density, high surface area, and a unique natural tubular structure, can be applied for water purification materials. We used an ultraviolet–visible spectrometer to measure UV absorbance of remaining organic pollutants, such as MB (a widely used textile industry waste, shown in Figure S7) and phenol (a common chemical industry waste, shown in Figure S8) in liquid to monitor the target pollutant degradation efficiency. Additionally, the photodegradation of MB can be mainly mediated by these oxygen species (eq 1).^71,72^
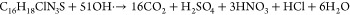
1Based on the previously reported photodegradation mechanism of ZnO nanomaterials toward methylene blue, a possible degradation pathway is presented in Figure S9.^73−75^ The initial oxidation process of the target dye pollutants can be caused by the successive attack from the hydroxyl radical, and/or by the hole transfer. Afterward, the intermediates were degraded to the final products by self-degradation or degradation via reactive oxidative species. CKZ samples (0.05 g), pure MB solution, MB solution with the same amount of kapok fibers, and MB solution with the same loading amount of ZnO powder were all tested under both dark environment and UV irradiation exposure (shown in Figures 3d, S7, and S10a–c). As discussed in the Supporting Information, it indicated the photodegradation ability of the ZnO powders and the limited absorption rates of the control groups in a dark environment. To eliminate the fiber absorption effect, the absorption amount in the dark environment was deducted. The net purification rates of CKZs are calculated in the Supporting Information and displayed in Figure 3d, which all showed over 80% MB degradation efficiency under only 0.5-h UV irradiation. As shown in Figure 3d, the net MB degradation rates of CKZ-120 min after 0.5, 1, 1.5, 2, 4, and 5 h of UV irradiation process are the highest among all of the CKZs. Particularly, the net MB degradation rates of CKZ-5 min, CKZ-10 min, CKZ-30 min, CKZ-60 min, CKZ-90 min, and CKZ-120 min after 5 h of UV irradiation process are 89.2, 90.6, 91.2, 91.6, 93.6, and 94.7%, respectively. Therefore, we stated that the photocatalytic performance of CKZ-120 min was the best among all CKZ samples. But in general, CKZ-30 min to CKZ-120 min had similar photodegradation efficiency, which suggests that CKZs can have good cost performance with only 30 min of growth time. The digital image shows the pristine MB solution and MB degradation results treated by CKZs after a 5-h UV irradiation process in Figure 3e. For further proof of catalytic efficiency on other organic pollutants, the degradation process of phenol was shown in Figure S8. Under UV irradiation, electrons in the valence band were excited and promoted to the conduction band leaving a hole behind. The electron–hole pairs can recombine or interact with other molecules.^76^ Thereinto, the holes may react with either other electron donors in solution, or hydroxide ions to form superoxide radicals or powerful oxidizing species.^77^ To further improve the UV-degradation performance, the doping method was applied by immersing the ZnO-loaded fibers into graphene oxide aqueous solution followed by heat treatment for reduction.

### 3.4 Improvement of Photodegradation Efficiency by the Doping Method

**Figure 4:**
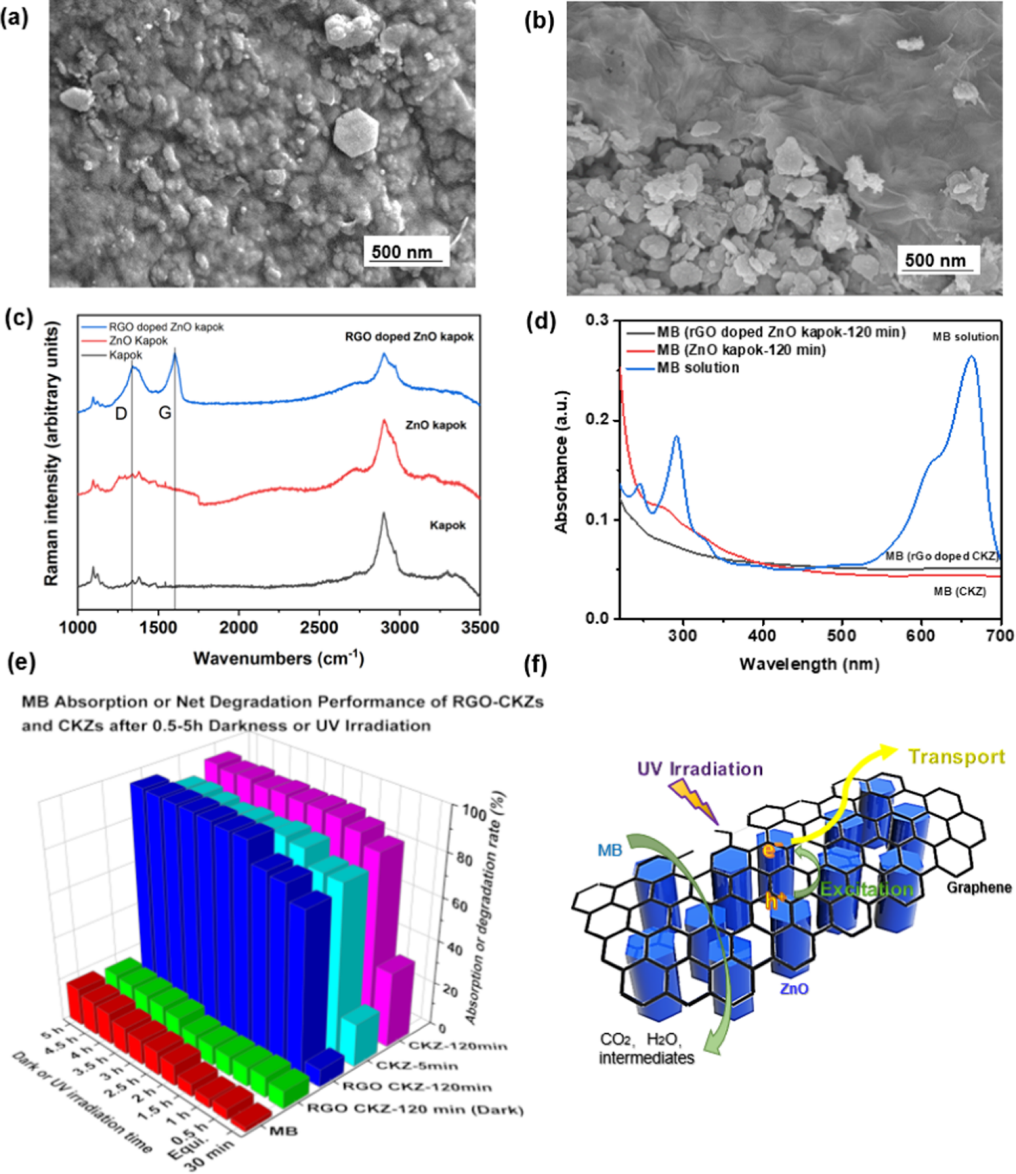
Characterization analysis, comprehensive analysis of MB degradation efficiency, and photocatalytic mechanism of RGO-doped CKZ photocatalysts. (a, b) SEM images of rGO-doped ZnO kapok fibers. (c) Raman spectra of pristine kapok fibers, ZnO-loaded kapok fibers, and rGO-doped ZnO kapok fibers. (d) Comparison of ultraviolet absorption spectra of MB solution treated with rGO-doped CKZ-120 min, CKZ-120 min, and pure MB solution after a 5-h UV irradiation process. (e) The absorption rate of rGO-doped CKZ-120 min after 5 h of darkness and net MB photocatalytic efficiency of rGO-doped CKZ-120 min, CKZ-5 min, CKZ-120 min, and pure MB solution samples after the 0.5, 1, 1.5, 2, 4, and 5 h of the UV irradiation process. (f) Schematic illustration of the photocatalytic mechanism of RGO-doped ZnO kapok photocatalysts.

The doping method can further improve the photodegradation activity by the modulation of dopants in the optoelectronic properties of zinc oxide, such as doping of ZnO with noble metals or advanced 2D materials.^78−83^ As the possibility of recombination of photogenerated electrons and holes can be reduced after doping, more activation sites are provided for UV degradation. Furthermore, efficient charge transfer between ZnO nanomaterials and conductive dopants leads to faster photocurrent generation and the optical absorption is presumably shifting toward the visible region by the doping method.^84,85^ Therefore, rGO with excellent conductivity and other advantages was chosen as the dopant and deposition material for the bi-composite. The heterostructure and morphology of CKZ with rGO (5 wt %) dopant are displayed in Figure 4. A smooth rGO layer wrapped over the fiber samples is shown in Figure 4a,b. Raman spectral patterns of the rGO-doped ZnO nanocomposite (Figure 4c) exhibits two characteristic main peaks: D band at 1361 cm^–1^ and G band at 1604 cm^–1^, arising from a breathing mode of κ-point photons of A_1g_ symmetry and the first-order scattering of E_2g_ photons of sp^2^ C atoms, respectively.^86,87^ As illustrated in Figures 4d,e and S10d, the rGO-doped CKZs with the same mass (but much smaller volume) of CKZs have shown obvious photodegradation activity, due to the low absorption effect. Notably, after only 2-h UV light irradiation, the net photodegradation efficiency of rGO-doped CKZ-120 min for MB was over 90%.

Figure 4f provides a possible mechanism of the photodegradation process of rGO-doped ZnO kapok hybrids. ZnO nanomaterials are excited by UV light to generate electrons that were transferring from the valence band to the conduction band and holes in the valence band. RGO on the fiber surface provides a smooth electron transport route in the rGO/ZnO structure, enhancing the separation of photogenerated electron–hole pairs. The photoexcited electrons can reduce the oxygen to generate the superoxide radicals (O^2-^), while the separated holes can react with water/OH^–^ ions to generate hydroxyl radicals, which would subsequently assist the degradation of organic pollutants. To investigate the generation of superoxide radicals and hydroxyl radicals, electron paramagnetic resonance (EPR) spectra of RGO/ZnO-120 min were recorded on a Bruker EMXmicro EPR spectrometer equipped with a Bruker ER4122-SHQ resonator, which can capture the radicals produced via light time catalyst trapped by DMPO in Figure S11. When RGO was doped onto the composite, ZnO nanocrystals shows several areas of defects and damage under the cover of RGO sheets. Additionally, the HRTEM image of the RGO/CKZ composite shows the lattice distance of RGO (∼0.34 nm) and interplanar spacing of ZnO (0.29 nm corresponding to the (100) crystal plane and 0.26 nm corresponding to the (002) crystal plane). Figure S12a shows the photoluminescence (PL) spectrum of RGO/CKZ-120 min with characteristic peaks at 538 and 364 nm, which correspond to the deep level emission (DLE) and near band edge (NBE) emission, respectively.^75^ As the DLE emission can be assigned to different interband defects within crystal structures and the NBE emission can originate from the free exciton recombination in the near band edge of ZnO nanostructure. Therefore, comparing CKZ-120 min, RGO/CKZ-120 min with more intense DLE emission in the PL spectrum can exhibit slightly higher defect concentration, which can be caused by larger excitable volume at hexagonal tips.^75^ RGO/CKZ with high defect density can also influence the band gap to enhance the degradation performance. Moreover, when doped with conductor materials, the band gap of ZnO can be narrowed and more excited electrons could reach the conduction band to accelerate the electron transfer process. As the electrons of the metal can be easily excited by light irradiation, it would also increase the final amount of free radicals, which are produced by the reaction between electron–hole pairs and oxygen or hydroxyl. Furthermore, it would reduce the recombination of charge carriers and widen the spectral range for UV degradation to improve the degradation efficiency.

### 3.5 Determination of Sample Durability

**Figure 5:**
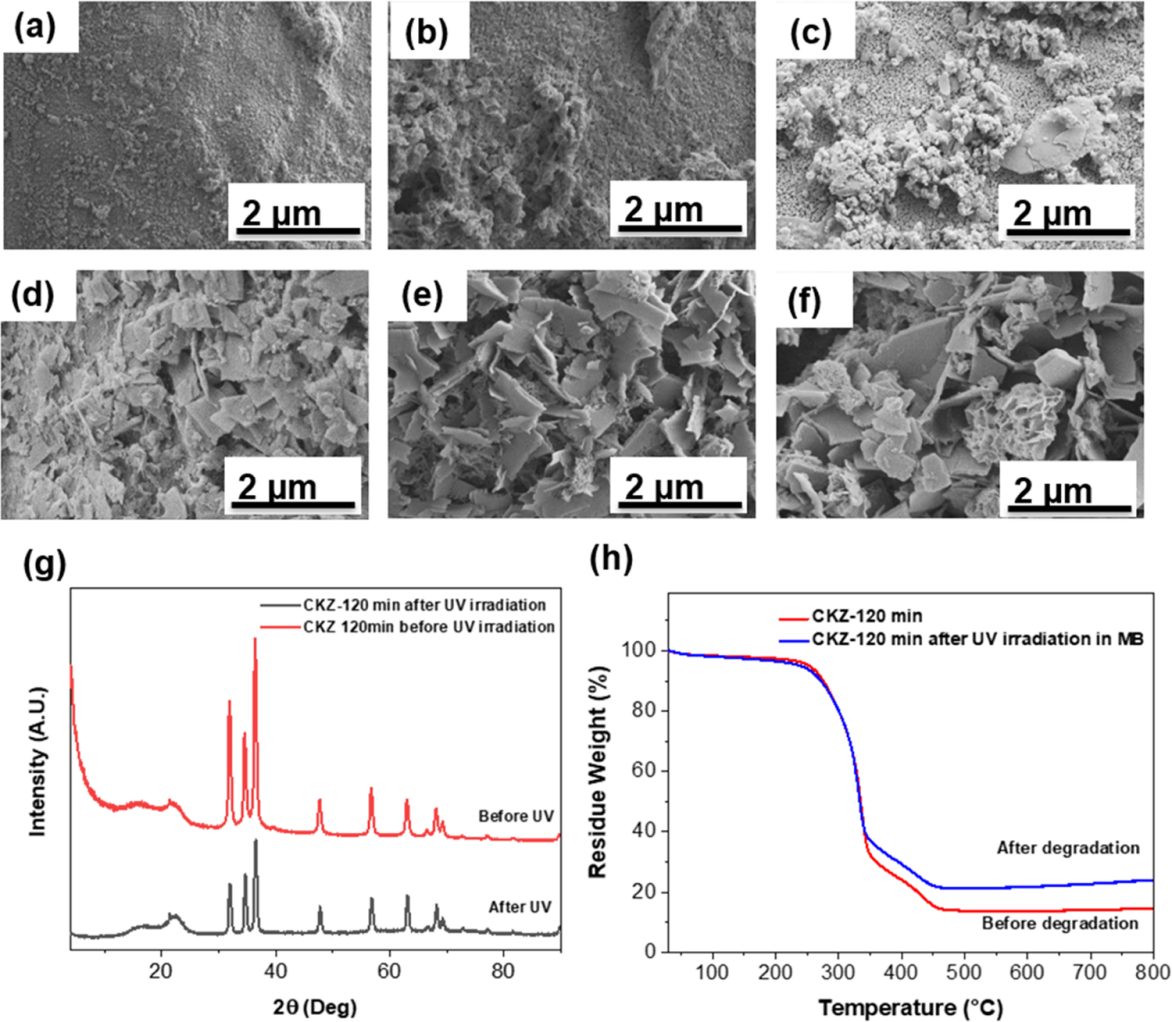
After a 5-h UV irradiation process, the morphology analysis of ZnO nanoparticles deposited on a kapok fiber substrate. Morphology of (a) CKZ-5 min, (b) CKZ-10 min, (c) CKZ-30 min, (d) CKZ-60 min, (e) CKZ-90 min, and (f) CKZ-120 min samples after 5-h UV irradiation. (g) XRD spectra of CKZ-120 min before and after the UV irradiation process. (h) TGA of CKZ-120 min before and after the UV irradiation process.

From the perspective of economic and recycling efficiency, the durability and reusability of our samples were explored by conducting 5 times repeated experiments. After the first 5 h of the UV-catalytic test, the morphologies of ZnO-loaded on the fibers are shown in Figure 5a–f, which suggests that the ZnO coating over the surface of kapok fibers suffered some corrosion. The hexagonal wurtzite-shaped ZnO nanocrystals underwent a disassembly process transforming into flakes again, compared with the growing process. The N_2_ adsorption–desorption results in Figure S4 also suggested that ZnO nanocrystals may suffer some disassembly process due to corrosion after UV irradiation with a lower specific surface area and smaller pore sizes, compared with ZnO before UV degradation. Therefore, the electroless deposition of ZnO nanocrystals can enhance the specific area of natural fibers with plenty of active sites, but these ZnO nanocrystals may suffer some damage on the crystal structures after degradation. However, the ZnO nanomaterials were still loaded on the fibers after an etching process during the irradiation, suggesting good durability of the supported UV degradation catalysts. In Figure 5g, the XRD analysis of the CKZ-120 min structure before and after the 5-h degradation process all show the wurtzite-type crystals. Although the intensity of ZnO (100) peak decreased, CKZ-120 min after UV irradiation shows a preferential orientation for the ZnO (101) peak. The decrease in the intensity suggested that the crystalline structure was damaged after treatment. However, no further change was observed in the peak width, indicating a relatively stable grain size. Figure 5h shows the thermal stability of CKZ-120 min before and after the MB degradation process from 30 to 800 °C. In the range from 30 to 100 °C, ∼5.6% weight of all samples was lost due to the evaporation of intercalated water molecules. Then, the TGA curves show a ∼78% weight loss in the range of 100–460 °C, which can be related to the elimination of the cellulose structures including the glycosidic bond cleavage and the formation of flammable gases. Thus, around 16.4% of the CKZ-120 min weight was due to the ZnO coated on the fiber surface. The contaminants previously absorbed onto the CKZ-120 min sample during the degradation can be the reason for the weight loss change.

**Figure 6:**
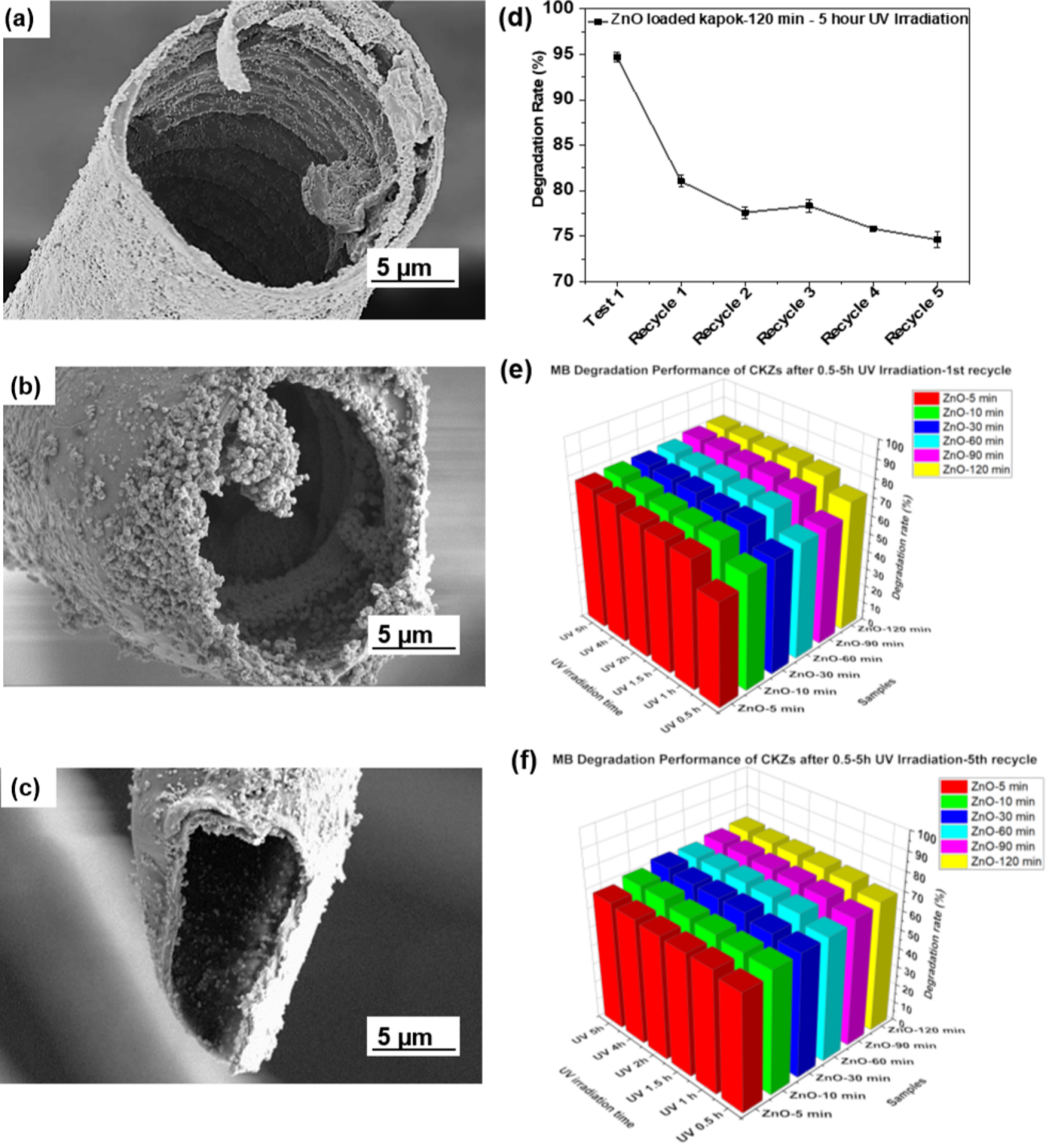
Recycling performance. (a) Cross-section morphology of CKZ-120 min, (b) after the 1st recycle process, and (c) after the 5th recycle process. (d) MB degradation rate of CKZ-120 min after 5 recycling processes with a UV irradiation time of 5 h in each process. (e) MB degradation rate after the 1st recycle process by CKZ-5 min, 10 min, 30 min, 60 min, 90 min, and 120 min, respectively, in the 0.5, 1, 1.5, 2, 4, and 5 h UV irradiation process. (f) MB Degradation rate after the 5th recycle process by CKZ-5 min, 10 min, 30 min, 60 min, 90 min, and 120 min, respectively, in the 0.5, 1, 1.5, 2, 4, and 5 h UV irradiation process.

Furthermore, ZnO nanocrystals are observed not only on the surface of the natural fibers but also inside of the fibers in Figure 6a–c. It is suggested that in this in situ method, zinc nitrate was dissolved and dissociated into Zn^2+^ ions, which could penetrate into the tubular cavity of kapok fibers. In the meantime, the natural hollow shape of fibers played a key role in the diffusion of the solution, due to capillarity, which significantly assisted the growth of ZnO nanocrystals on the inner wall of fiber substrates. According to the diffusion effect in microtubes, the concentration of solution within microtubes was different from that beyond microtubes. Therefore, the structure of ZnO was different on the inner and exterior walls of fibers. Due to outstanding penetrability of UV light and the ultrathin properties of the kapok fibers, UV light can penetrate the fiber wall of kapok and more activation sites can be excited on the inner wall of kapok fibers to improve the photodegradation efficiency (Figure S1g). More importantly, the ZnO nanocrystals attached to the inner wall of fibers can prevent exfoliation during the recycling cleaning process, which can assist in improving reusability (Figure 6b,c). The TGA curves (Figure S13f) of CKZ-120 min before and after washing cycles showed a typical curve structure of fiber loaded with ZnO materials, suggesting good durability of the ZnO-loaded composite. As shown in Figures 6d–f and S13a–e, the photodegradation performance of MB after 5 reuse cycles on CKZ UV degradation catalysts were around 78, 74, 70, 70, and 68% after 5 h of UV irradiation, respectively. Particularly, CKZ-120 min (Figure 6d) has been proved to have a relatively stable photodegradation efficiency (around 75%) even after 5 recycling processes. It is suggested that the unique hollow structure of the bio-substrate can significantly improve the efficiency and prolong the service life of the catalysts. Additionally, to illustrate the degradation performance more comprehensively, the results of the RGO/CKZ cycle experiment are shown in Figure S12b. The results suggested good sustainability of RGO/CKZ samples for water purification application.

## 4 Conclusions

Zinc oxide (ZnO) crystals loaded on ultrathin, hollow kapok fibers were successfully fabricated via the proposed curcumin bio-interfacial entanglement method facilitated by simple electroless deposition (ELD). Curcumin was successfully functionalized on the surface of the kapok fiber, providing abundant seeding sites for the growth of ZnO. The ELD method effectively controlled the formation of intensive and firm ZnO nanocrystals on the outer and inner walls of the hollow kapok fibers. The ZnO-loaded kapok fiber as hybrid UV degradation catalysts showed high catalytic efficiency and structural stability for ultraviolet photodecomposition of organic pollutants, methylene blue (MB) and phenol. The ZnO-loaded kapok fiber with 120-min prolonged ELD time was found to be the optimum ultraviolet catalyst and the ZnO catalysts can provide efficiency of over 80% after 0.5 h of ultraviolet irradiation for the photodegradation reaction. The as-prepared ZnO UV-degradation catalysts can be recycled and reused after 5 cycles with a catalytic efficiency of around 70% after a 5-h irradiation process. Additionally, by decorating reduced graphene oxide on ZnO-kapok ultraviolet catalyst, the degradation efficiency of over 90% was achieved in only 2 h of ultraviolet irradiation. Overall, by making full use of the “offcut” natural fibers, the durable ZnO-kapok heterogeneous catalyst underlines the advantage of this water purifier in industrial pollution control in an economical and environmentally friendly way. In addition, the demonstrated unique curcumin bio-interfacial entanglement method facilitated by a simple electroless deposition opens new research avenues in functionalization and modification of fiber materials as advanced materials, particularly for the development of highly durable sustainable absorbent and supported catalysts.

## Supporting Information Available

SEM images, EDX analysis of Pd treatment, UV–vis spectra of MB or phenol after UV irradiation, UV–vis spectra of MB after each recycling process, and degradation analysis (PDF)
Video S1: Dewaxed kapok fibers modified with curcumin (AVI)

## Supplementary Material

am1c21554_si_001.pdf

am1c21554_si_002.avi
