# Supplementary material for: Entangled ZnO on Ultrathin Hollow Fibers for UV-Aided Pollutant Decomposition
Source: ACS Appl Mater Interfaces. 2022 Feb 21;14(8):10769–81. doi: 10.1021/acsami.1c21554 (PMC9098110; doi:10.1021/acsami.1c21554)
Supplement: Supplementary file 1 — am1c21554_si_001.pdf [file am1c21554_si_001.pdf]

Supporting Information

Entangled ZnO on Ultra-Thin Hollow Fibres for UV-Aided Pollutant Decomposition

AUTHOR NAMES

Xi Wang [a], Shaojun Xu [b, c], Evelyn Chalmers [a], Xiaogang Chen [a], Yong Liu [d] and Xuqing Liu [a]*

AUTHOR ADDRESS

[a] Department of Materials, School of Natural Sciences, the University of Manchester, Oxford Road, Manchester, M13 9PL, U.K.

[b] UK Catalysis Hub, Research Complex at Harwell, Didcot, OX11 0FA, UK.

[c] Cardiff Catalysis Institute, School of Chemistry, Cardiff University, Cardiff, CF10 3AT, UK.

[d] School of Textile, Tiangong University, No. 399 Bin Shui Xi Road, Xi Qing District, Tianjin 300387, P.R. of China.

CORRESPONDING AUTHOR

*Dr. Xuqing Liu - Department of Materials, B47, Sackville Street Building, the University of Manchester, Oxford Road, Manchester, M13 9PL, U.K. E-mail: xuqing.liu@manchester.ac.uk Tel: +44 01613064125

KEYWORDS

Curcumin interfacial functionalization, Electroless deposition, Zinc oxide, Natural hollow fibre substrate, Wastewater

1. Dewaxing process

Kapok fibres were dewaxed by the enzyme (lipase). 2 g enzyme was dissolved into 100 mL phosphate buffer and 1 g kapok fibres were added into the solution stirred at 38 °C for 10 hours. Then, the pretreated fibres were rinsed 5 times in deionized water (DI water) and dried in an oven at 40 °C for 8 hours.

2. Preparation of graphene oxide in lab.

Graphene oxide (GO) was produced from graphite powder using a modified Hummers method. 54 ml of sulfuric acid and 6 ml of phosphoric acid were mixed and stirred for 10 mins. 0.45 g of graphite powder was added into the above mixture while stirring. Then 2.64 g of potassium permanganate (K_2MnO_4) was added into the solution slowly and carefully and then stirred for 6 hours until the colour changed to dark green. To eliminate left KMnO4, 1.35 ml of hydrogen peroxide was dropped slowly into the solution with stirring for 10 mins. Then the mixture was left for cooling down. 20 ml of hydrochloric acid and 60 ml of DI water was added into the solution and centrifuged by Eppendorf Centrifuge 5430R at 8000 rpm for 10 mins. Then the supernatant was decanted away and the residuals were cleaned three times following above method. The GO solution was then dried in oven at 90 °C for 24 hours to get GO powder for later application.[1]

3. Experimental section of EPR test

In the experimental section, all solvents and reagents were of analytic grade and were purchased from Sigma-Aldrich (Dorset, UK) and used without further purification. Sample mixtures were transferred into 1.3mm outer diameter (0.8mm inner diameter) silica tubes and then inserted into a 4mm outer diameter (3mm inner diameter) quartz EPR tube. The position of the sample in the resonator was optimised from measurements on a TEMPO standard solution.

Uniform UV irradiation at 365 nm was achieved using a Thorlabs M365L3 mounted LEDREF1 collimated using a Thorlabs SM2F32-A adjustable collimation adaptor. A 5x5mm offcut of the woven fabric was submerged and placed flat down into the bottom of glass vial, aliquots of the surrounding DMPO mixture were taken at 15 minutes and 30 minutes and transferred immediately into the capillary tubes.

X-band (9.4GHz) continuous wave EPR measurements were carried out on a Bruker EMXmicro EPR spectrometer equipped with a Bruker ER4122-SHQ resonator. Spectrometer settings were: microwave power 23dB (1.1 mW), modulation amplitude 0.5 G, sweep time 60 s receiver gain 30dB with an average microwave frequency of 9.86GHz.

4. Results and discussion

Characterization of kapok fibres samples.

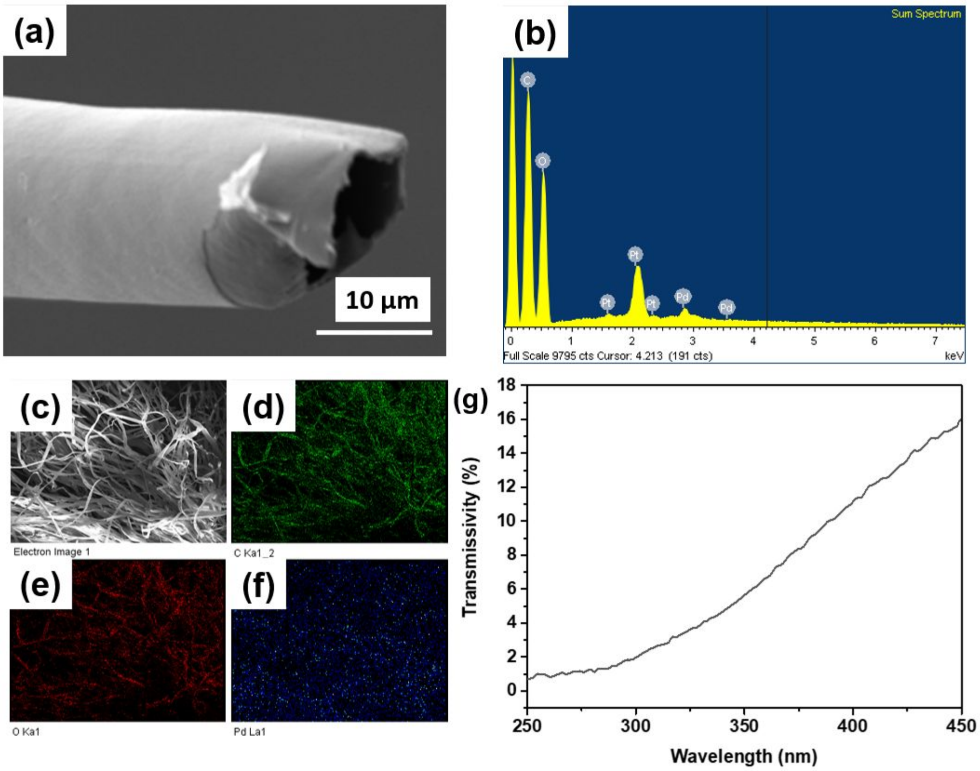

Figure S1. (a) SEM images of untreated kapok fibres. EDX analysis and corresponding elemental mapping of Pd on kapok fibres with curcumin. (b) EDX spectrum and (c,d,e,f) EDX element mapping. (g) UV transmittance spectrum of kapok fibres.

After curcumin modification, the palladium (Pd) ions might be chelated with the phenol groups of curcumin for dense ZnO electroless deposition. The palladium peak in the EDX spectra in **Figure S1** indicates the presence of Pd on the kapok surface and 0.105 wt% Pd is measured. Also, it is apparent from the EDX images that palladium was distributed throughout the fibre area. Thus, the immobilization of catalysts on the fibres for ELD is ready. To prove the penetrability of UV light, the UV transmittance test was conducted. The UV transmittance spectra of pristine kapok (**Figure S1g**) have shown a poor UV blocking performance. In this case, the UV light, not utilized by the ZnO nanomaterials on the outer walls of fibre would have a chance to penetrate the fibre structure and be utilized by the ZnO nanocrystals loaded

on the inner fibre walls. In addition, from the SEM images, it can be observed that kapok fibres have ultra-thin fibre walls (around 1 um). Therefore, these CKZ samples would make the most of the UV irradiation to enhance the photodegradation efficiency during the process.

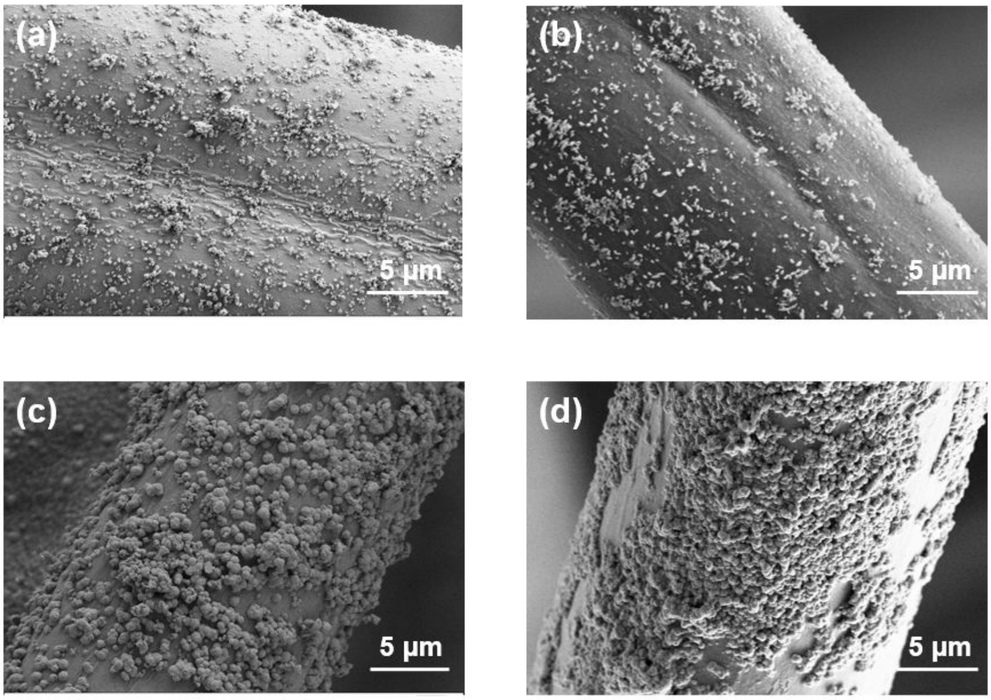

Figure S2. The morphologies of control groups. ZnO deposited on the fibres without curcumin modification (a) Kapok ZnO-10 min, (b) Kapok ZnO-30 min, (c) Kapok ZnO-90 min, and (d) Kapok ZnO-120 min.

To further illustrate the influence of kapok modified with curcumin on the growth of ZnO, we prepared some control samples with ZnO grown on the kapok substrate without curcumin pretreatment.

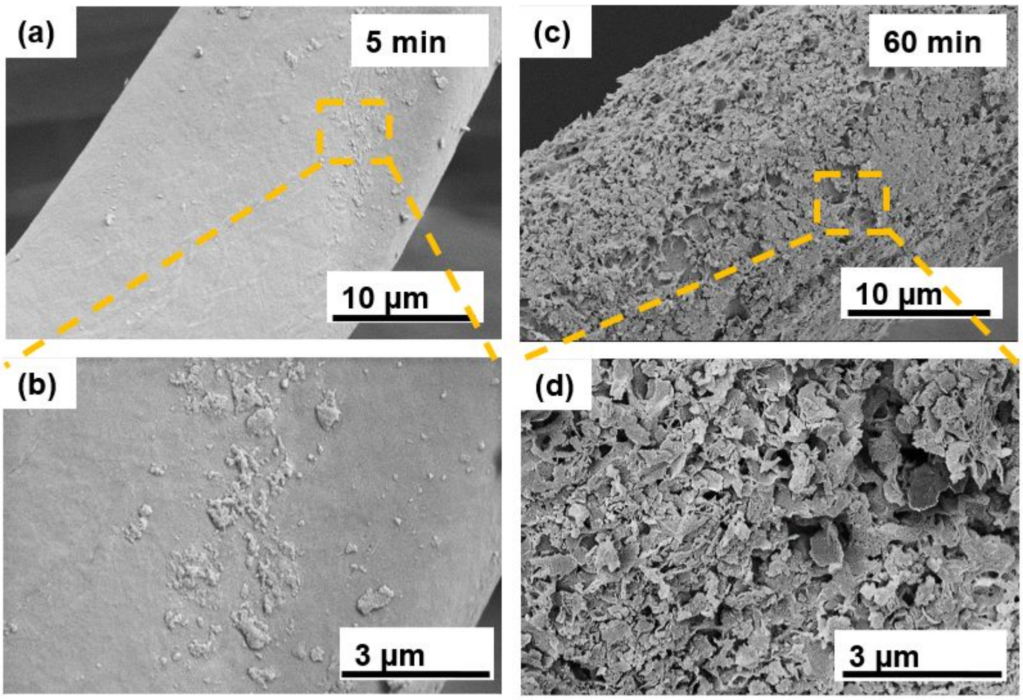

Figure S3. Morphology analysis of ZnO nanocrystals obtained after (a-b) 5 min and (c-d) 60 min prolonged time in scanning electron microscopy.

Figure S3 shows the SEM images of ZnO nanocrystals on fibres illustrating the morphology change during the growing process with the time expanded from 5 min to 60 min with a magnification of 10,000.

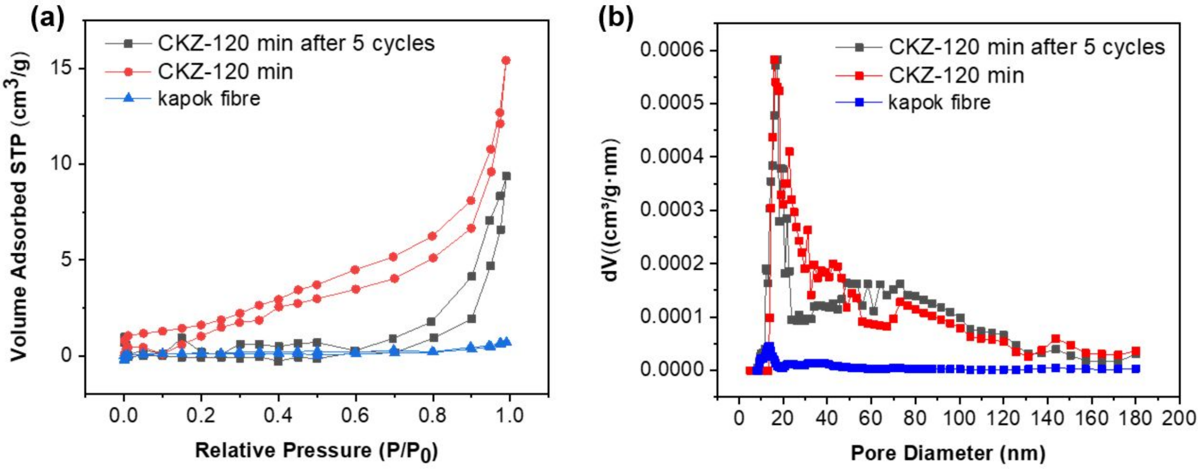

Figure S4. (a) N2 adsorption–desorption isotherms of pristine kapok fibres, CKZ-120 min, and CKZ-120 min after 5 cycle tests and (b) the corresponding pore size distribution curves of the pristine kapok fibres, CKZ-120 min, and CKZ-120 min after 5 cycle tests.

The deposition of ZnO nanocrystals throughout the hollow fibres can prevent the general agglomeration and provide a highet specific surface area with activation sites. Compared with the raw kapok fibre, CKZ-120 min showed a type IV N_2 adsorption/desorption isotherm with the H3 adsorption hysteresis loops.[2] The predominantly mesopores structures of CKZ-120 min can be deduced from the abrupt adsorption at the high relative pressure area. The Brunauer-Emmett-Teller (BET) surface area of pristine kapok fibre, CKZ-120 min and CKZ-120 min after 5 cycles were 0.355, 5.712, and 1.254 m^2/g, respectively, indicating the improvement on the specific surface area with the uniform nanocrystal layers on fibre substrate. The well-distributed nanostructured ZnO has provided a high specific surface area with many activation sites for the degradation process, which can lead to a distinctive improvement of UV degrading ability after the ELD process. However, as shown in **Figure S4**, the main pore size distribution of CKZ-120 min was between 10 to 80 nm while that of CKZ-120 min after 5 cycles was between 10 to 30 nm. The results suggested that ZnO nanocrystals may suffer some disassembly process due to corrosion after UV irradiation with a lower specific surface area and smaller pore sizes, compared with ZnO before UV degradation. Therefore, the electroless deposition of ZnO nanocrystals can enhance the specific area of natural fibres with plenty of active sites but may suffer some damage to the crystal structures after degradation.

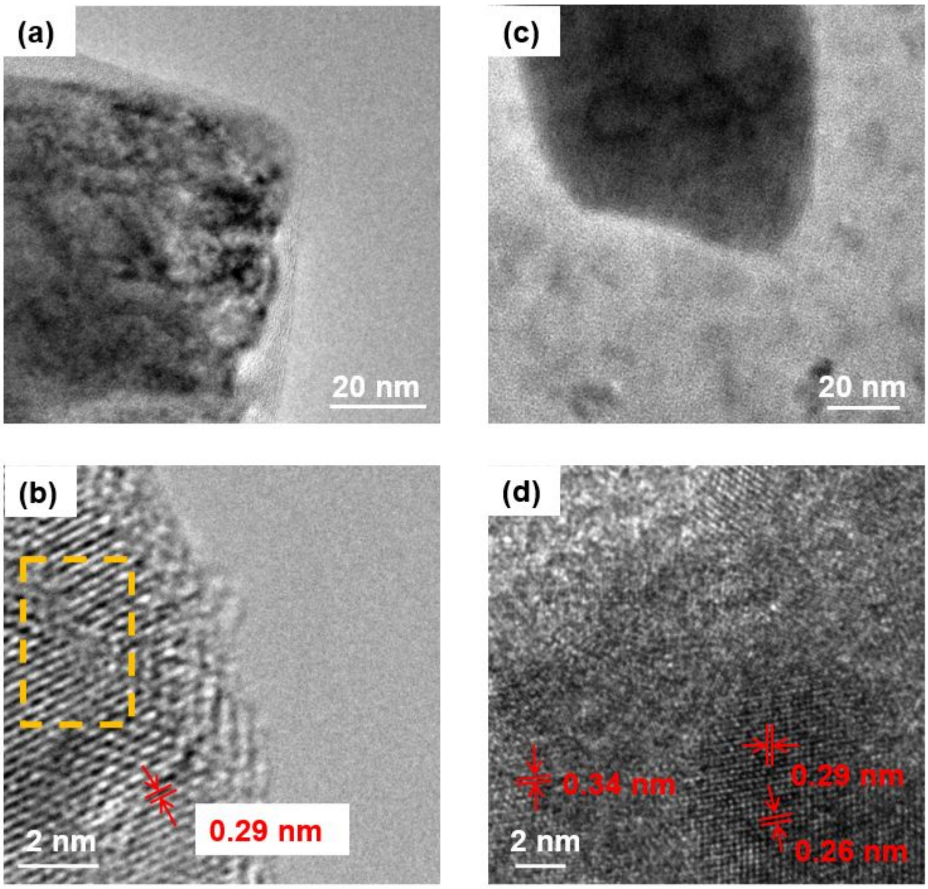

Figure S5. HRTEM images of (a-b) CKZ-120 min and (c-d) RGO-CKZ-120 min.

The TEM and high-resolution TEM (HRTEM) images of CKZ-120 min with structural flaws are shown in **Figure S5**. The crystallinity of ZnO can be reduced as a result of oxygen vacancies reducing anion-cation coordination.

When RGO was doped to the composite, ZnO nanocrystals shows several areas of defects and damage under the cover of RGO sheets. Additionally, the HRTEM image of RGO/CKZ composite shows the lattice distance of RGO (approximately 0.34 nm) and interplanar spacing of ZnO (0.29 nm corresponding to the (100) crystal plane and 0.26 nm corresponding to the (002) crystal plane).

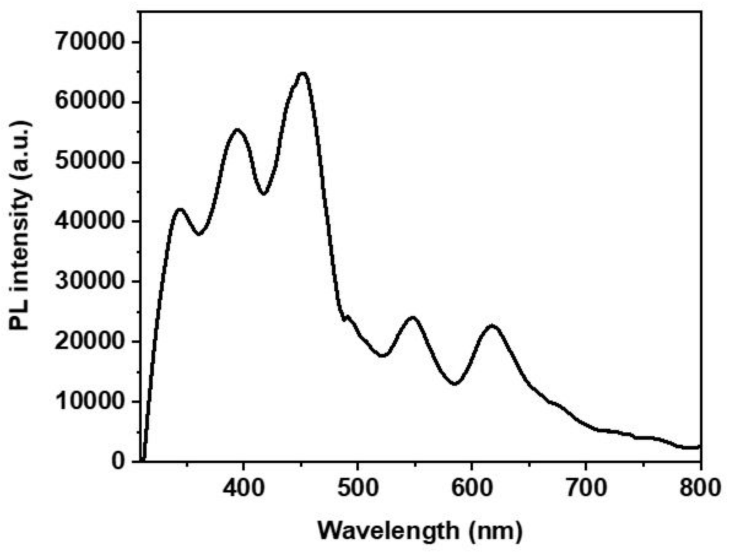

Figure S6. Photoluminescence (PL) spectrum of CKZ-120 min.

To further demonstrate the presence of oxygen holes on the surface of nanoscaled ZnO, photoluminescence (PL) spectroscopy was utilised to analyse the oxygen vacancy in the wavelength range of 300 to 800 nm using a 310 nm excitation wavelength.

UV degradation performance

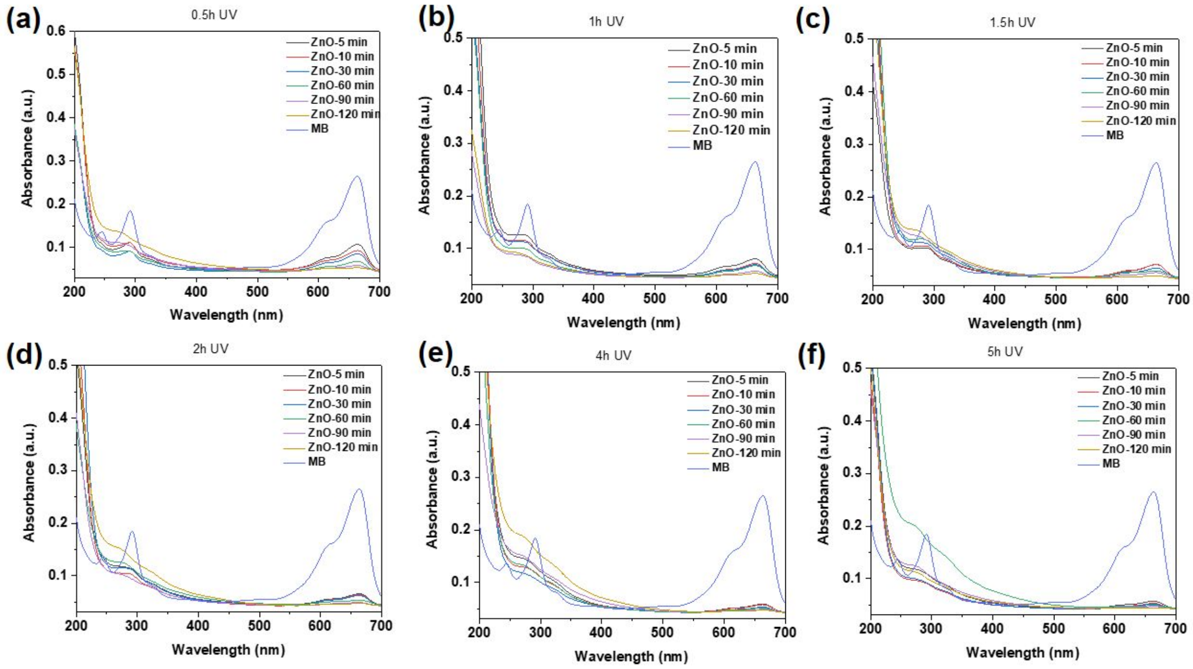

Figure S7. UV-vis spectra analysis of a) Contrast group MB and MB treated by CKZ with different prolonged time after 0.5 hour, b) 1 hour, c) 1.5 hours, d) 2 hours, e) 4 hours, and f) 5 hours UV irradiation.

The UV absorption spectra in **Figure S7** show the MB concentration changes during the photodegradation process of CKZs, in which the characteristic absorption peaks of MB at 663 nm, 292 nm, and 246 nm were all decreasing as irradiation time increased.

Furthermore, standard concentration curves (**Figure S7**) at 663nm were used to determine the concentration of MB solution after different periods of UV irradiation. The efficiency (degradation rate R) of the fibre-based UV degradation catalyst was calculated by the following formula:

$$R(\%) = \frac{C_0 - C_t}{C_0} \times 100\% \quad (1)$$

C_0 ($mg \cdot L^{-1}$) is the initial dye concentrate value of methylene blue solution, and C_t ($mg \cdot L^{-1}$) is the concentration value of methylene blue solution after t min of UV irradiation.

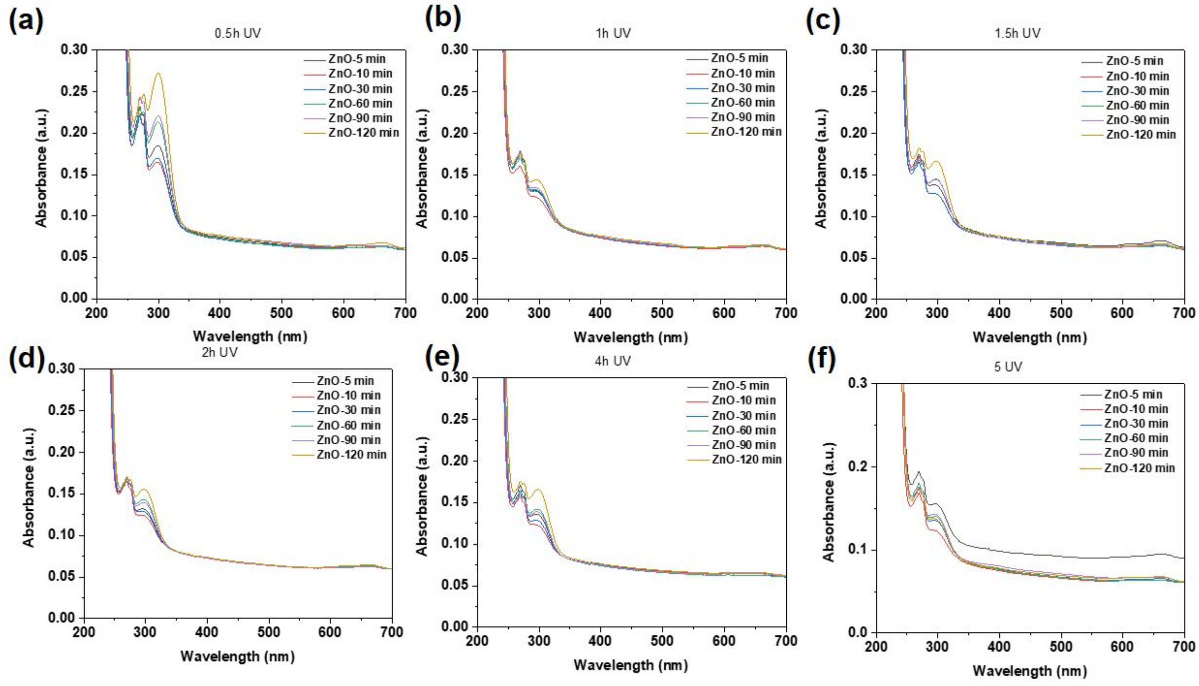

Figure S8. UV-vis spectra analysis of a) phenol (100 mg/L) treated by CKZ with different prolonged time after 0.5 hour, b) 1 hour, c) 1.5 hours, d) 2 hours, e) 4 hours, and f) 5 hours UV irradiation.

UV degradation process of ZnO-kapok fibre-supported UV degradation catalysts for phenol is shown in **Figure S8** after 5-hour UV light irradiation, which indicates that the UV degradation catalysts are not only able to degrade MB, but other organic pollutants.

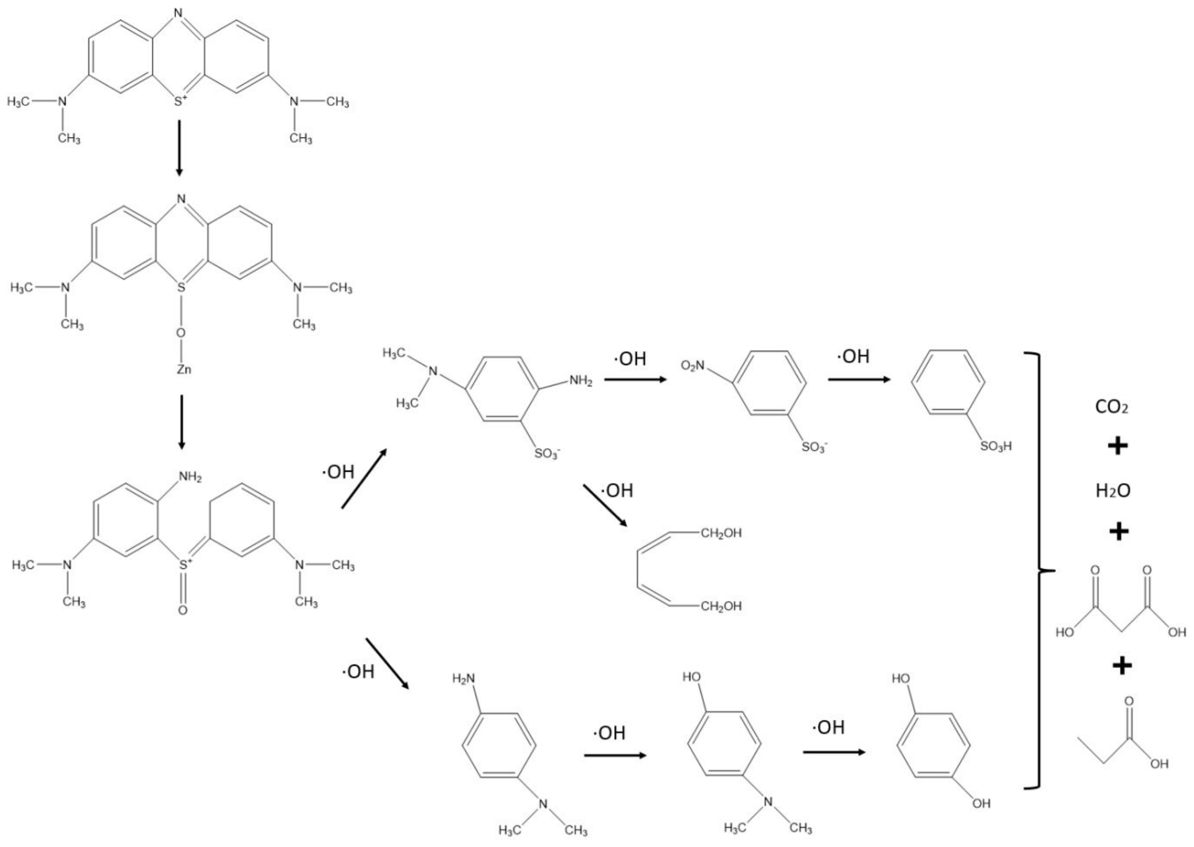

Figure S9. A plausible degradation pathway of methylene blue

Based on the previously reported photodegradation mechanism of ZnO nanomaterials towards methylene blue, a possible degradation pathway is presented in **Figure S9.**[3-5]

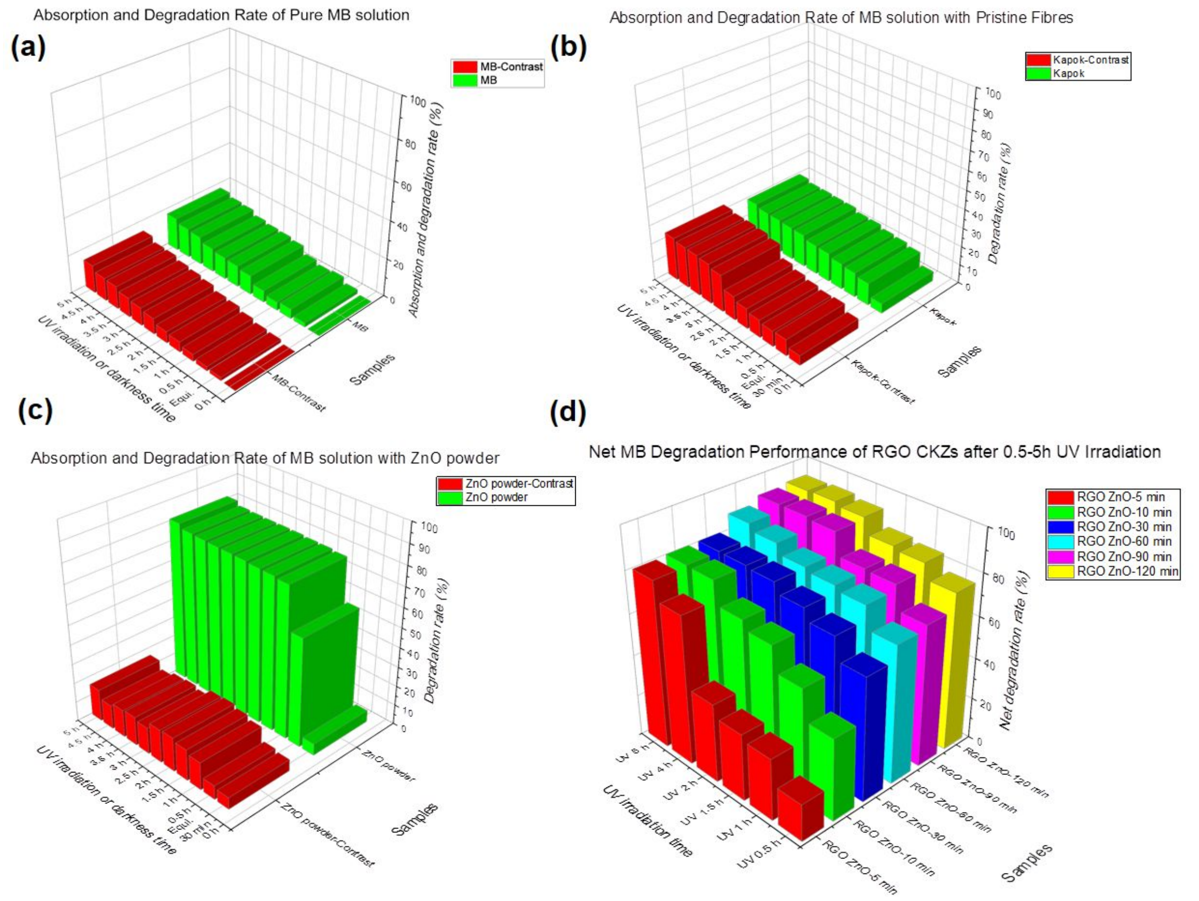

Figure S10. Absorption and degradation rate of a) Pure MB solution, b) MB solution with cotton fibres, and c) MB solution with ZnO powder after 0.5, 1, 1.5, 2, 4, 5 hours of dark or UV irradiation process. (d) The net degradation rate of MB treated by rGO doped CKZ with different prolonged times after 0.5, 1, 1.5, 2, 4, 5 hours UV irradiation process.

The degradation or absorption rates of Pure MB solution, MB solution with kapok fibres of the same amount and MB solution with ZnO powder of the same amount were applied as the control groups (shown in **Figure S10)**. 0.05 g of the CKZ samples were tested, while pure MB solution, MB solution with kapok fibres of the same amount and MB solution with ZnO powder of the same loading amount were all applied as the control groups to be tested under both dark

environment and UV irradiation exposure (shown in **Figure 3d and S10)**. Specifically, the absorption rates of pure MB solution, MB solution with kapok fibres and MB solution with ZnO powder under 5-hour darkness were around 17%, 20% and 17 %, respectively in **Figure S10**. Furthermore, compared with the 3 control groups after 5 hours of UV irradiation, the degradation rates were 18%, 19% and 80 % respectively, which illustrated the photodegradation ability of the ZnO powders. Also, the absorption rates of the 3 control groups in a dark environment have suggested that the raw fibres can only offer limited absorption ability. In addition to this, the absorption rates of CKZs after 30 min dark absorption-equilibration experiment were around 25% shown in **Figure 3d**, which can be due to the surface modification of ZnO to improve the absorption ability. Hence, to achieve excellent water purifying results, both absorption and photodegradation ability are required. As kapok fibres can provide both the high surface area and excellent absorption ability, the fibre-based ZnO hybrid structure is a promising material for water treatment.

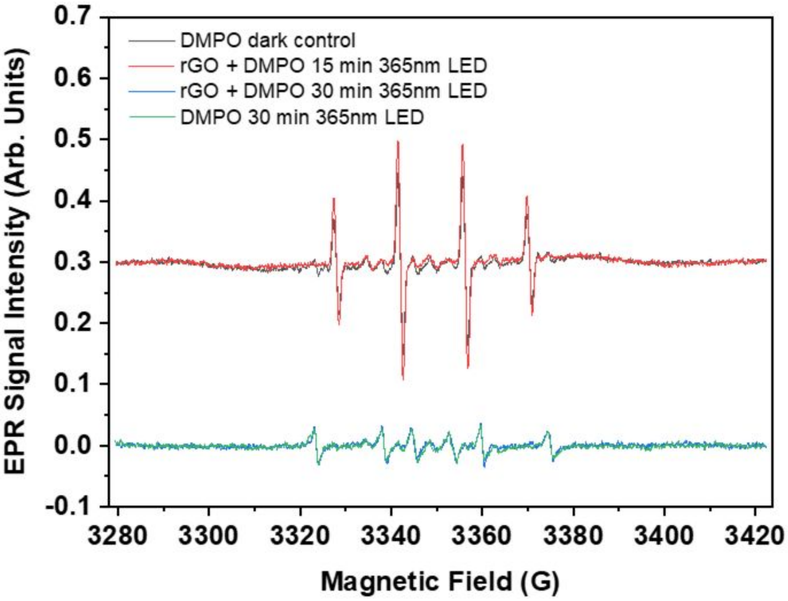

Figure S11. Electron spin resonance (ESR) experiments of RGO/ZnO-120 min.

Superoxide radicals and hydroxyl radicals have both been proved significant active free radicals for photodegradation of organic pollutants in water.[6] DMPO spin-trapping studies under aqueous conditions report the presence of intrinsic DMPO/•OH arising from the slow hydrolysis of DMPO.[7] We did observe examples of this particularly evident in the ZnO spin trapping experiments. **Figure S11** illustrates the generation of the characteristic 4 line 1:2:2:1 intensity pattern of the DMPO/•OH radical adduct when the RGO/CKZ-120 min was irradiated in the presence of a water/DMPO mixture. Control experiments on this DMPO/water mixture exhibit an intrinsic level of DMPO/•OH and carbon centered radical adducts in the dark state. The intensity of each component increases after irradiation which we attribute to the hydrolysis of the DMPO discussed above. Upon irradiation, the DMPO/•OH radical adduct component dominates the spectra as expected if further DMPO/•OH radical adducts are generated. It suggested that UV irradiation is the key element

for the generation of these free radicals, which would improve the degradation of organic dyes.

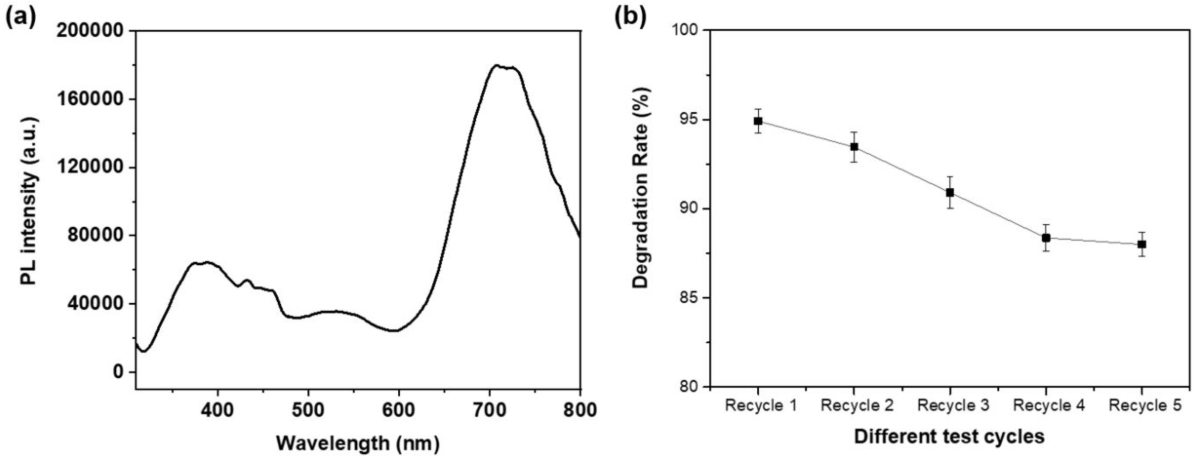

Figure S12. (a) Photoluminescence (PL) spectrum of RGO/CKZ-120 min and (b) MB degradation rate of RGO-CKZ-120 min after 5 recycling processes with 5 hours UV irradiation time in each process.

Figure S12a shows the photoluminescence (PL) spectrum of RGO/CKZ-120 min with characteristic peaks at 538 and 364 nm, which are corresponding to the deep level emission (DLE) and near band edge (NBE) emission, respectively.[5] To illustrate the degradation performance more comprehensively, the results of RGO/CKZ cycle experiment are shown in **Figure S12b**. The results suggested good sustainability of RGO/CKZ samples for water purifying application

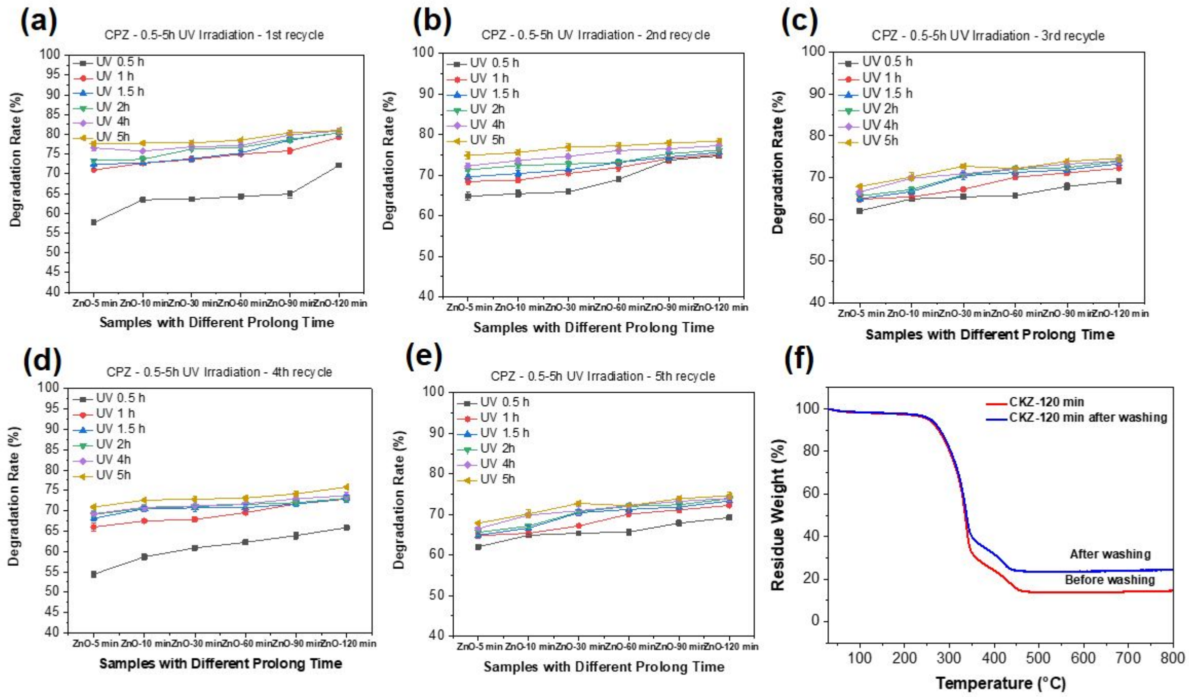

Figure S13. Comprehensive analysis of MB degradation rate a) after 1st recycle test, b) after 2nd recycle test, c) after 3rd recycle test, d) after 4th recycle test, and e) after 5th recycle test. (f) TGA of CKZ-120 min before and after washing cycles.

Figure S13 displays the UV degradation performance of MB after 5 reusing cycles by CKZ UV degradation catalysts after 5-hour UV irradiation, respectively. The TGA curves of CKZ-120 min before and after washing cycles showed the typical curve structure of fibre loaded with ZnO materials, suggesting the good durability of ZnO-loaded composite.

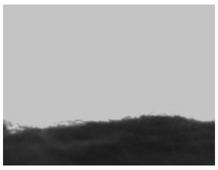

Video S1. Contact angle test of dewaxed kapok fibres modified with curcumin (30 s).

REFERENCE

1. Chen, J.; Yao, B.; Li, C.; Shi, G., An improved Hummers method for eco-friendly synthesis of graphene oxide. *Carbon* **2013**, 64, 225-229.
2. Wang, J.; Xia, Y.; Dong, Y.; Chen, R.; Xiang, L.; Komarneni, S., Defect-rich ZnO nanosheets of high surface area as an efficient visible-light photocatalyst. *Applied Catalysis B: Environmental* **2016,** *192*, 8-16.
3. Trandafilović, L. V.; Jovanović, D. J.; Zhang, X.; Ptasińska, S.; Dramićanin, M., Enhanced photocatalytic degradation of methylene blue and methyl orange by ZnO: Eu nanoparticles. *Applied Catalysis B: Environmental* **2017,** *203*, 740-752.
4. Lin, J.; Luo, Z.; Liu, J.; Li, P., Photocatalytic degradation of methylene blue in aqueous solution by using ZnO-SnO2 nanocomposites. *Materials Science in Semiconductor Processing* **2018,** *87*, 24-31.
5. Reimer, T.; Paulowicz, I.; Röder, R.; Kaps, S. r.; Lupan, O.; Chemnitz, S.; Benecke, W.; Ronning, C.; Adelung, R.; Mishra, Y. K., Single step integration of ZnO nano-and microneedles in Si trenches by novel flame transport approach: whispering gallery modes and photocatalytic properties. *ACS applied materials & interfaces* **2014,** *6* (10), 7806-7815.
6. He, J.; Zhang, Y.; Guo, Y.; Rhodes, G.; Yeom, J.; Li, H.; Zhang, W., Photocatalytic degradation of cephalexin by ZnO nanowires under simulated sunlight: Kinetics, influencing factors, and mechanisms. *Environment international* **2019,** *132*, 105105.
7. Ranguelova, K.; Mason, R. P., The fidelity of spin trapping with DMPO in biological systems. *Magnetic Resonance in Chemistry* **2011,** *49* (4), 152-158.
